# The origins of dengue and chikungunya viruses in Ecuador following increased migration from Venezuela and Colombia

**DOI:** 10.1186/s12862-020-1596-8

**Published:** 2020-02-19

**Authors:** Irina Maljkovic Berry, Wiriya Rutvisuttinunt, Rachel Sippy, Efrain Beltran-Ayala, Katherine Figueroa, Sadie Ryan, Abhinaya Srikanth, Anna M. Stewart-Ibarra, Timothy Endy, Richard G. Jarman

**Affiliations:** 1 Viral Diseases Branch, Walter Reed Army institute of Research, Silver Spring, MD USA; 2 Institute for Global Health and Translational Science, SUNY Upstate Medical University, Syracuse, NY USA; 3 Department of Medicine, Technical University of Machala, Machala, El Oro Ecuador; 4 Quantitative Disease Ecology and Conservation (QDEC) Lab, Department of Geography, University of Florida, Gainesville, FL USA; 5 Emerging Pathogens Institute, University of Florida, Gainesville, FL USA; 6 Department of Medicine, SUNY Upstate Medical University, Syracuse, NY USA; 7 Department of Montevideo, InterAmerican Institute for Global Change Research (IAI), Montevideo, Uruguay; 8 Department of Microbiology and Immunology, SUNY Upstate Medical University, Syracuse, NY USA

**Keywords:** Dengue, Chikungunya, Ecuador, Venezuela, Colombia, Dissemination

## Abstract

**Background:**

In recent years, Ecuador and other South American countries have experienced an increase in arboviral diseases. A rise in dengue infections was followed by introductions of chikungunya and Zika, two viruses never before seen in many of these areas. Furthermore, the latest socioeconomic and political instability in Venezuela and the mass migration of its population into the neighboring countries has given rise to concerns of infectious disease spillover and escalation of arboviral spread in the region.

**Results:**

We performed phylogeographic analyses of dengue (DENV) and chikungunya (CHIKV) virus genomes sampled from a surveillance site in Ecuador in 2014–2015, along with genomes from the surrounding countries. Our results revealed at least two introductions of DENV, in 2011 and late 2013, that initially originated from Venezuela and/or Colombia. The introductions were subsequent to increases in the influx of Venezuelan and Colombian citizens into Ecuador, which in 2013 were 343% and 214% higher than in 2009, respectively. However, we show that Venezuela has historically been an important source of DENV dispersal in this region, even before the massive exodus of its population, suggesting already established paths of viral distribution. Like DENV, CHIKV was introduced into Ecuador at multiple time points in 2013–2014, but unlike DENV, these introductions were associated with the Caribbean. Our findings indicated no direct CHIKV connection between Ecuador, Colombia, and Venezuela as of 2015, suggesting that CHIKV was, at this point, not following the paths of DENV spread.

**Conclusion:**

Our results reveal that Ecuador is vulnerable to arbovirus import from many geographic locations, emphasizing the need of continued surveillance and more diversified prevention strategies. Importantly, increase in human movement along established paths of viral dissemination, combined with regional outbreaks and epidemics, may facilitate viral spread and lead to novel virus introductions. Thus, strengthening infectious disease surveillance and control along migration routes and improving access to healthcare for the vulnerable populations is of utmost importance.

## Background

Arboviruses, especially dengue and yellow fever, are thought to have a long history of presence in the Americas, where numerous suspected outbreaks have been recorded since the 1600s [1]. Upon discovery of the *Aedes aegypti* (*Ae. aegypti*) mosquito as the main vector for transmission of yellow fever by Walter Reed in year 1900, attempts to eliminate the vector and the diseases it carried were undertaken in the Americas [2, 3]. Initially the campaigns were highly successful, achieving elimination of *Ae. aegypti* by 1962 in many Latin American countries, including South American countries of Brazil, Peru, Ecuador, Colombia and Paraguay [3, 4]. However, the arboviral vector was not eliminated in other countries such as Venezuela and Cuba, and this, in combination with the steady deterioration of the vector elimination programs over time, resulted in re-expansion of *Ae. aegypti*. As the vector returned, so did the arboviral diseases.

Ecuador and other South American countries have since experienced a steady increase in the number of arbovirus related infections, especially dengue [1, 5, 6]. Today dengue is hyper-endemic in some regions of Ecuador, and previously absent severe forms of dengue disease are on the rise [6, 7]. Dengue is not the only mosquito-borne disease to reemerge in the Americas. Chikungunya virus (CHIKV) has been suggested to have caused outbreaks in the Caribbean and the Gulf of Mexico in the 1820s, which was then followed by a long absence of the virus from this region [8, 9]. CHIKV reemerged in 2013 in the Caribbean and quickly spread to the countries of North, Central and South America [10–12]. In Ecuador, the first cases of CHIKV were reported at the end of 2014, and in an outbreak in the southern part of the country, 43% of suspected dengue cases were confirmed to actually be acute CHIKV infections (only 28% were confirmed to be acute DENV) [6]. Following the invasion of CHIKV, an arbovirus novel to the region, Zika virus (ZIKV), was identified in the Americas [13]. It reached Ecuador in early 2016, when the first autochthonous transmission was documented in the country. In recent years, all three arboviruses (DENV, CHIKV and ZIKV) have co-circulated in Ecuador and its neighboring countries.

The spread of arboviruses is affected by population susceptibility and driven by human and vector movement. Increased global prevalence and invasion of new areas by *Ae. aegypti* and *Ae. albopictus*, the two main arboviral vectors, has resulted in an increase in arbovirus presence throughout the world [14–16]. Human movement has been suggested to be the major contributor to the spread of viruses between countries and across long distances, mainly through international travel and migration, but also through internally displaced populations [15, 17–22]. Human migration specifically has recently been on a substantial rise in South America. Due to the socioeconomic and humanitarian conditions in Venezuela, more than 3 million people have been estimated to have departed this country so far, with the majority of the exodus occurring after 2014 [23]. Unsurprisingly, the neighboring countries of Colombia, Ecuador, Chile, Argentina and Peru have taken in more than half of all the Venezuelan refugees [24]. Venezuela, due to the collapse of its health care system, has also experienced an increase in infectious diseases such as malaria, measles, diphtheria, dengue, chikungunya, Zika and others [25, 26]. The massive human movement from this country has thus led to concerns of considerable increases in infectious disease spillover and spread across this region of South America. There is currently little known about the regional implications of this migration on infectious disease spread, including countries that serve as major migratory routes and population recipients, such as Ecuador. In Ecuador, an active surveillance site in the harbor city of Machala revealed the first characterized CHIKV outbreak in the country, as well all four serotypes of dengue, all simultaneously circulating in 2014–2015. In this study, we use the most prevalent Ecuadorian DENV (DENV1 and DENV2) and CHIKV genomes from this time period to determine the origins and the time of emergence of these arboviral strains in Ecuador, and we describe the role of the neighboring countries in the dissemination and spread of these pathogens throughout the region.

## Results

**Fig. 1:**
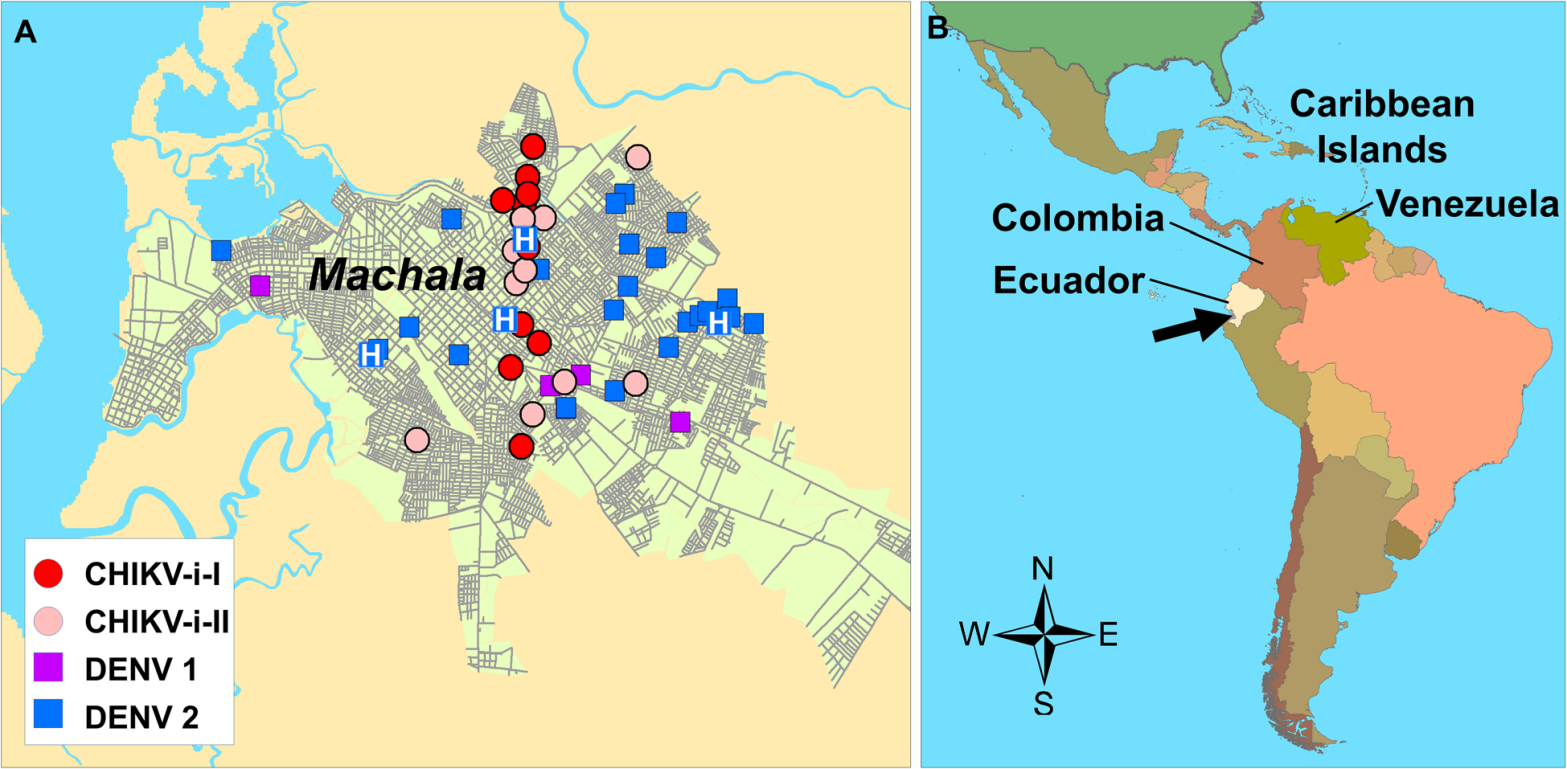
Study site location of *A. Machala*, Ecuador, showing locations of sequenced cases of DENV1 and DENV2, and the two introductions of Chikungunya (CHIKV-i-I, CHIKV-i-II); clinics are shown by ‘H’, with the central hospital on the same site as the central clinic location; and B. Machala (black arrow) on the coast of Ecuador, on the western coast of South America

A total of 40 novel arboviral genomes were sequenced from samples collected in the southern city of Machala, Ecuador, population: 280,694, latitude: 3°15′S, longitude: 79°57′W. The samples were collected throughout the metropolitan area of Machala (Fig. 1). The sequenced genomes included two DENV serotypes (1 and 2), that belonged to the genotype V and Asian American, respectively. CHIKV genomes belonged to the Asian lineage, and are the first genomes of CHIKV to be reported from the country of Ecuador (Additional file 1).

### Venezuela and Colombia as main origins of DENV1 and DENV2 dissemination and introductions into Ecuador

**Fig. 2:**
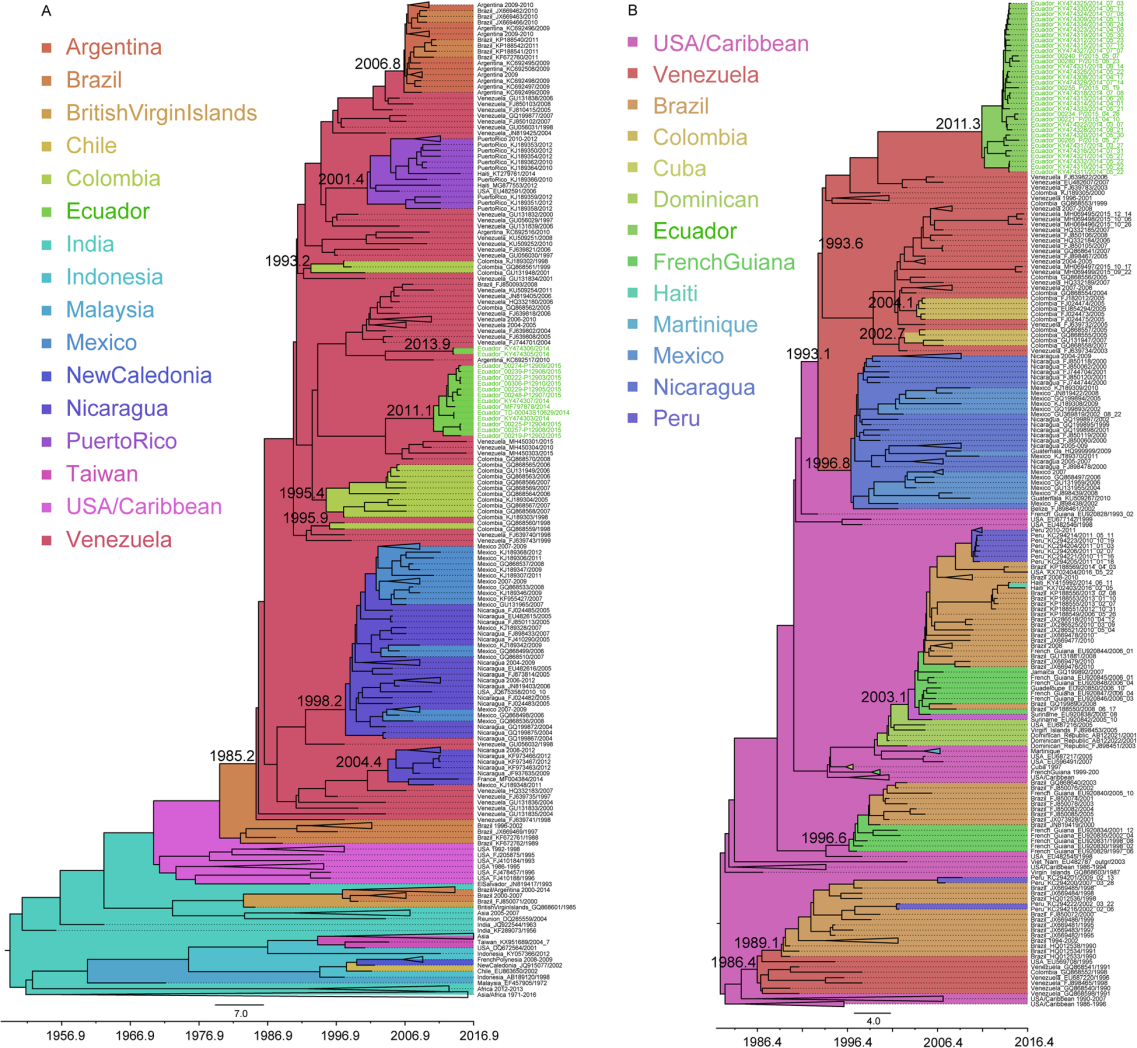
Large dataset MCC trees of **a**) DENV1 (genotype V, American sublineage) and **b**) DENV2 (Asian American genotype). Taxa from Ecuador are color coded in green. Location origins are colored in the tree according to the legend. Times of the most recent common ancestors discussed in the text are noted next to the respective ancestor nodes

DENV1 samples collected in 2014 and 2015 in Machala, Ecuador, were found in two separate clusters in the maximum clade credibility (MCC) tree, indicating two separate introductions of the virus spreading in this region (Fig. 2a). The first introduction occurred in the beginning of 2011 (2011.1; HPD: 2009.5–2012.3), and this virus persisted for at least 4.5 years in Ecuador. The second introduction occurred at the end of 2013/beginning of 2014 (2013.9; HPD: 2013.5–2014.1) and this virus strain had only persisted in Machala for approximately 6 months before being detected. The two different strains of DENV1 were circulating simultaneously in Machala in 2014, while in 2015 only one of the strains was sampled. Ancestral viruses from both introductions were suggested to have originated in Venezuela, although the exact paths of virus dissemination remain unclear. Phylogeographic analyses indicate that Venezuela seems to have played an important role in the dissemination of DENV1 throughout South and Central/North America, with many of the ancestral viruses seeding the DENV1 introductions into the South and Central/North American countries originating from here (Fig. 2a). This includes Colombia, where DENV1 was introduced in 1993 and twice in 1995 (mid and late year); Nicaragua, with introductions in 1998 and again in mid-2004; Puerto Rico, with introduction in mid-2001; Argentina, with DENV1 introduction at the end of 2006; and Ecuador with two DENV1 introductions in 2011 and again in late 2013. All these introductions resulted in successful and sustained spread and persistence of the virus in these countries. Several additional introductions, supported by single genomes in the tree, into Colombia, Brazil and Argentina, with ancestral viruses originating in Venezuela, were also observed. Although the tree suggests that DENV1 was originally seeded into Venezuela in 1985 from Brazil, the support for this is low, indicating missing data.

Two full genome datasets (a smaller one consisting of ~ 100 genomes and a larger one consisting of ~ 300 genomes) and one E gene dataset per DENV serotype were used in Bayesian analyses to investigate the impact of data size and data type on the temporal and spatial tree inferences. Interestingly, while full genome Bayesian Evolutionary Analysis by Sampling Trees (BEAST) analyses of both small (BEASTFGD1.small, Additional file 2) and large (BEASTFGD1.large, Fig. 2a) datasets for DENV1 produced concordant results pertaining to the majority of introductions, some discrepancies were observed. In the BEASTFGD1.small (Additional file 2) MCC tree, the country of origin of the first introduction into Ecuador was suggested to be Colombia rather than Venezuela. However, this location probability was lower compared to the one from the large dataset. In general this was true for most location probabilities when comparing the small and the large dataset inferences (Additional file 3). E gene analyses of DENV1 also confirmed most viral introductions estimated by the full genome analyses, but showed discrepancies in the introductions into Ecuador, indicating Colombia as the source of both introductions (Additional file 4). Since more genomes were available for the E gene analyses, these also suggested viral dissemination from Venezuela into Mexico, Barbados, Guyana, as well as indicated that the dissemination from Venezuela into Brazil resulted in further spread and circulation of this DENV1 strain between Brazil, Argentina, Paraguay and Uruguay.

In addition to DENV1, DENV2 was frequently sampled in both 2014 and 2015 in Machala, Ecuador. DENV2 genomes were found in a monophyletic cluster in the MCC tree indicating a single introduction of this virus into Machala (Fig. 2b). However, this cluster was clearly separated into two distinct sub-clusters, of which one suggested a recent major expansion of this variant in Machala, as indicated by very short branches and a most recent common ancestor (MRCA) existing in late 2013. The two sub-clusters coalesced into a common ancestor that was estimated to have originated in Venezuela, and entered Ecuador by 2011 (2011.3; HPD: 2010.5–2012). Our results suggest involvement of Venezuela and Brazil in the dissemination of DENV2 in this region. Ancestral DENV2 viruses existing in Venezuela were introduced into Brazil in 1989, into Nicaragua in the mid-1996, into Colombia in mid-2002 and again in 2004, and into Ecuador by 2011. Several additional single genome introductions into Colombia were also observed. Interestingly, more recent samples from Venezuela, collected in 2015, were found more closely related and sharing a major cluster with genomes from Colombia, and not Ecuador. Instead, the current Ecuadorian DENV2 variant was most closely related to DENV2 sampled in Venezuela in 2007. The MRCA of the 2014–2015 Ecuadorian genomes and the 2015 genomes from Venezuela existed in 1993 indicating early divergence and circulation of at least two different DENV2 strains in Venezuela, followed by eventual introduction of one of the strains into Ecuador by 2011. Despite Peru and Ecuador being direct neighbors, their DENV2 genomes had no close genetic relationship. Instead, ancestral DENV2 viruses existing in Brazil were introduced into Peru at several time points. In addition, DENV2 strains were disseminated from Brazil to Haiti, USA and French Guiana. We also notice at least 4 major introductions (in 1986, 1993, 1996, and 2003) of DENV2 into South America resulting in successful establishment and spread of DENV2 sublineages across the continent. Two of these sublineages still persisted at the time of this study.

Full genome BEAST analyses of both small (BEASTFGD2.small, Additional file 5) and large (BEASTFGD2.large, Fig. 2b) datasets for DENV2 produced concordant results pertaining to their respective genetic relationships, TMRCAs (time of the most recent common ancestor) and the locations of viral origins. The only differences were in their estimated location probabilities (Additional file 3). The smaller dataset in general produced lower location posterior probabilities, while the larger dataset had high confidence probabilities for viral location origins (Additional file 3). In addition, and unsurprisingly, the larger dataset was able to detect more viral introductions, such as an additional dissemination of DENV2 from Brazil to Peru. E gene analyses of DENV2, however, did not produce results that were completely concordant with full genome analyses. For DENV2 E gene (Additional file 6), the origin of Ecuadorian genomes was estimated to Colombia and this country was also estimated to have disseminated the virus at several time points into Venezuela (all posterior probabilities > 0.97) and once into Nicaragua. E gene analyses also suggested viral dissemination from Venezuela into Colombia (2012) and Peru (1998), and from Peru into Ecuador (1999). As observed for DENV1 E gene results, DENV2 also indicated viral spread between Brazil, Paraguay and also Bolivia.

### Several independent introductions of CHIKV into Ecuador from the Caribbean/Central America

**Fig. 3:**
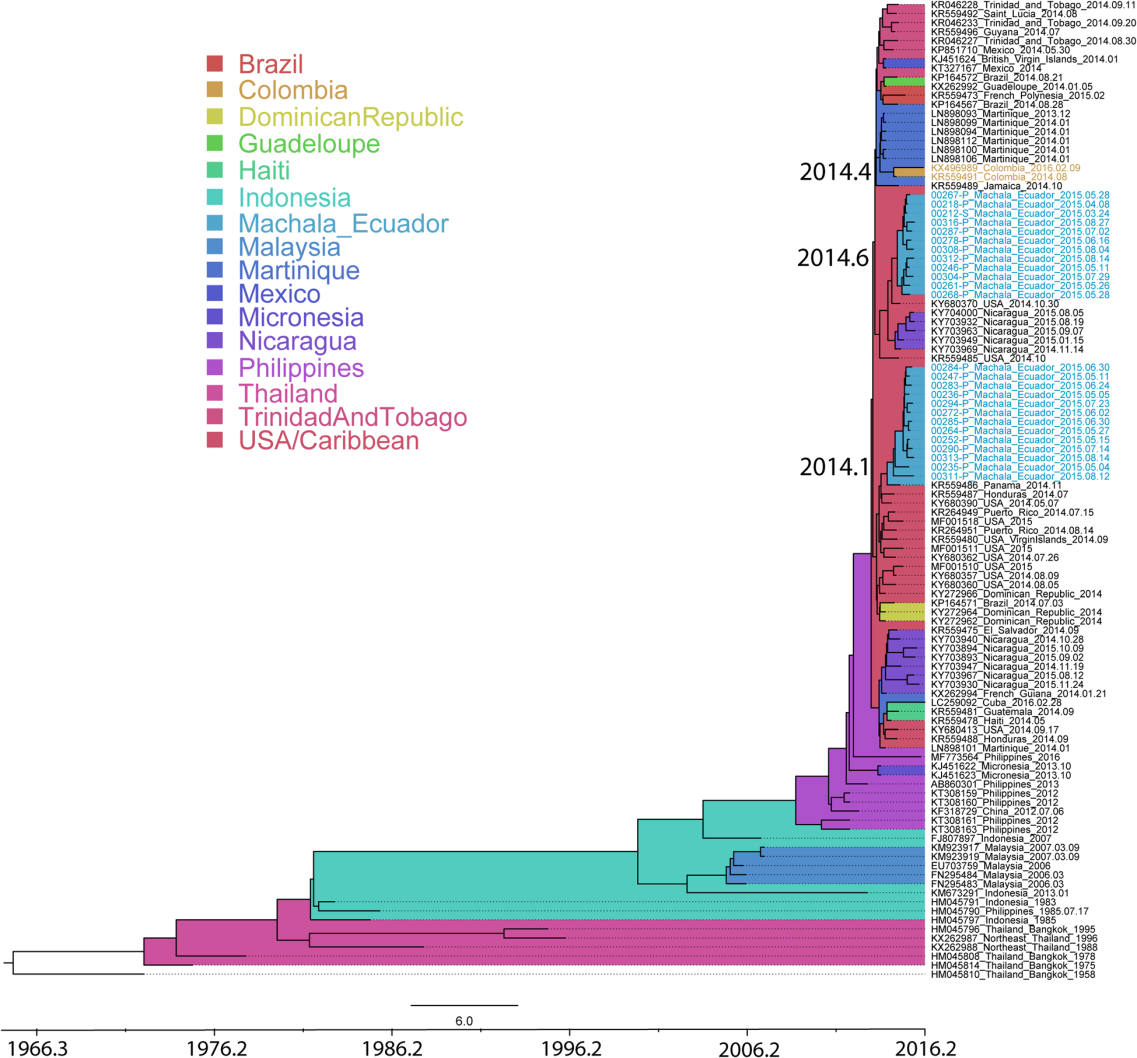
MCC tree of CHIKV Asian lineage. Taxa from Ecuador are color coded in light blue. Location origins are colored in the tree according to the legend. Times of the most recent common ancestors discussed in the text are noted next to the respective ancestor nodes

CHIKV was circulating in Machala, Ecuador in 2015 (Additional file 1), and the genomes from this outbreak were found in two separate monophyletic clusters in the Asian genotype MCC tree, indicating two independent introductions of CHIKV into Ecuador (Fig. 3). Both introductions originated from the Caribbean/USA and were found to have occurred in an interval of 5–6 months, with the first introduction in early 2014 (2014.1; HPD: 2013.7–2014.5) and the second one in mid-2014 (2014.6; HPD: 2014.4–2015.0). Even though the surveillance site was active in 2014, the first CHIKV was sampled in March 2015, indicating a period of silent CHIKV transmission in this region of 8 months (second introduction) to 1.2 years (first introduction). Viruses from the two introductions did not make distinct geographic clusters in the city of Machala, rather, they were dispersed across the city, indicating simultaneous circulation of the two CHIKV strains across the same area (Fig. 1). Interestingly, CHIKV in Colombia was not closely related to either of the Ecuadorian clusters. The virus was disseminated into Colombia from Martinique in mid-2014 (2014.4; HPD: 2014.2–2014.6).

CHIKV genomes from Ecuador were screened for reported mutations that have previously been associated with virus phenotype change in the vector, such as enhanced viral fitness, transmission and infection of the mosquito. As all Asian lineage CHIKV strains, the viruses from Ecuador had the E1:A98T residue change and the E1:K211E change (Table 1). E1:A98T restricts positive selection of the *Ae. albopictus*-adaptive E1:A226V substitution [28, 29]. E1:K211E, in combination with E1:V264A and in the presence of E1:A226 wild type, has been described to enhance fitness of the virus in *Ae. aegypti,* and has recently been observed in CHIKV outbreaks in several regions of the world [30, 32]. These results show that CHIKV from Ecuador had not acquired all the mutations that could possibly increase its fitness to the main vector in Ecuador, *Ae. aegypti*.

**Table 1:** CHIKV amino acid mutations associated with phenotype change in vector

Protein	Amino acid substitution	Phenotype	Asian lineage	Asian lineage Ecuador cluster only
E1	A226V	Enhanced infection of *Ae .albopictus* [27]	A	A
E1	A98T	Restricts positive selection of the *Ae. albopictus*-adaptive E1-A226V substitution [28, 29]	**T**	**T**
E1	K211E	Enhanced fitness in *Ae aegypti* in background of E1-226A [30]	**E**	**E**
E2	V264A		V	V
E2	K233E/Q	Enhanced infection of *Ae. albopictus* [28]	K	K
E2	K234E	Enhanced infection of *Ae. albopictus* [28]	K	K
E2	K252Q	Enhanced infection of *Ae. albopictus* – secondary mutation [28]	K	K
E2	R198Q	Enhanced infection of *Ae. albopictus*, synergistic with E3-18F in background of E1-226V [28]	R	R
E2	L210Q	Enhanced infection of *Ae. albopictus*, synergistic with E3:18F in background of E1:226V [28, 31]	L	L
E3	S18F	Enhanced infection of *Ae. albopictus*, synergistic with E2:R198Q [28]	S, **F**	S

Bold and underline - vector adaptive residues

### Immigration and case count data relative to arbovirus introductions

**Fig. 4:**
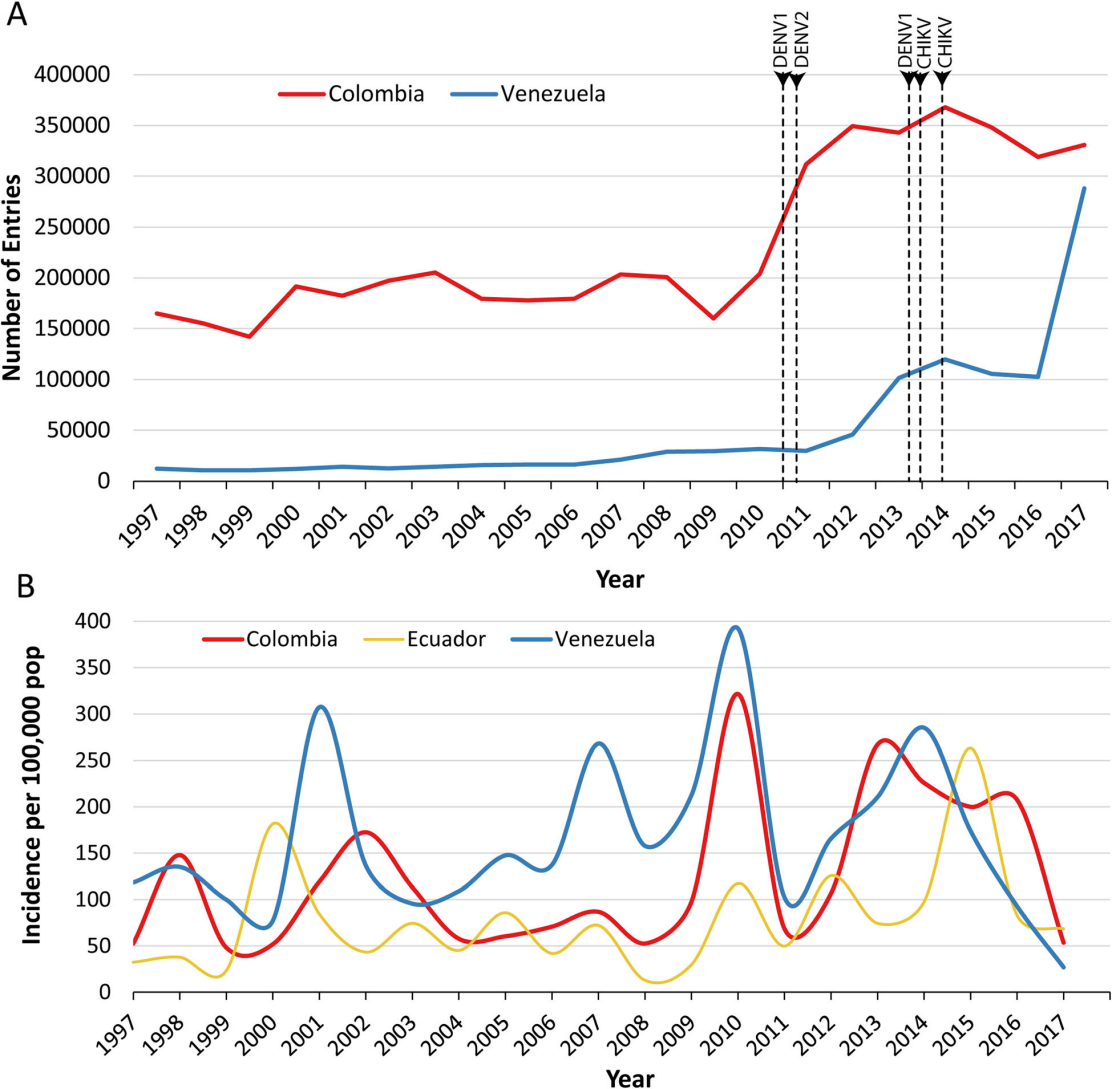
**a** Annual number of Venezuelan and Colombian citizens entering Ecuador. DENV1, DENV2 and CHIKV introductions are noted as vertical lines. CHIKV lines are black, and DENV lines are color coded based on their country of origin association. **b** Dengue incidence per 100,000 population in Colombia, Venezuela and Ecuador (data from PAHO)

Immigration data from the National Institute for Statistics and Census in Ecuador (INEC) [33] between 1997 and 2017 indicated a sharp rise of Colombian citizens entering Ecuador, starting 2009–2010, and peaking around 2014. The influx of Venezuelan citizens into Ecuador was steady until 2007, when an increase of 30% was observed compared to the previous year. Another sharp increase started in 2012, by 54% compared to the year before, and by 2014 the number of immigrants had increased by 305% since 2011. In 2017, the influx of Venezuelan citizens had increased by 900% compared to 2011 (Fig. 4a). The 2011 DENV1 and DENV2 introductions into Ecuador, and the 2013/2014 DENV1 and CHIKV introductions, both followed an increase of Colombian and Venezuelan citizens entering the country (Fig. 4a). However, these introductions also followed an increase of dengue cases in Colombia and Venezuela (Fig. 4b) [34].

## Discussion

Ecuador has in recent years experienced increases of arboviral diseases, from increased numbers of dengue infections to introductions of novel viruses - chikungunya and Zika - and their sustained spread in the country. The same pattern has also been observed in other nearby Andean countries in South America, such as Colombia and Venezuela. In addition, Venezuela’s growing social, political and economic crisis has resulted in hyperinflation, poverty, and a collapse of basic support systems, such as health care and vector control to combat the infectious diseases that are common in this area of the world. This has led to an additional increase of infections within Venezuela, with diseases such as malaria, measles, dengue, chikungunya and Zika on the rise [35]. As the citizens are fleeing from the increasingly harsh living conditions in Venezuela, the concerns of spread and further rise of infectious diseases in the neighboring countries have been growing [36]. In Ecuador, an increase of malaria cases has recently been observed, with the parasite reemerging in regions previously declared malaria-free [37]. This includes regions in the south of the country, near the Ecuador-Peru border, which are located along the migration route from Venezuela, through Colombia and Ecuador, and into Peru. The samples analyzed in this study came from the southern coastal city of Machala. This tropical city is an important sentinel site, as it is a major port located along the Pan American Highway, near the Ecuador-Peru border. DENV is hyperendemic in Machala, and is transmitted by the *Ae. aegypti* mosquito vector (*Ae. albopictus* has not been detected) [6]. By analyzing the arboviral genomes collected in 2014–2015 from the active surveillance site in Machala, we attempt to understand the dissemination of these pathogens throughout the region, including their introductions into Ecuador and their connections to the pathogens from Venezuela and Colombia.

Our results of dengue spread indicate several introductions into Ecuador, with DENV1 being introduced in at least two different time points. Both DENV1 and DENV2 introductions were strongly correlated to the genomes circulating in Venezuela and Colombia, and the results implied possible dissemination of these viruses from these two countries into Ecuador. The introductions were estimated to have occurred by 2011 and by the end of 2013 (DENV1), and by the spring of 2011 (DENV2). Ecuador experienced a sharp increase of migration into the country from Colombia starting in 2009, and the migration of Venezuelans started increasing already in 2007 with a sharp increase in 2012 and then again in 2017. The introduction of DENV1 and DENV2 strains in 2011 preceded the start of the mass population movement from Venezuela, however, it followed the increase of the influx of Colombian citizens into the country. The introduction of DENV1 into Ecuador in late 2013/early 2014 happened after the increase of the influx of Colombian and Venezuelan citizens in 2009 and 2012, respectively. Interestingly, DENV case count data indicate that both the 2011 and the 2013/2014 introductions into Ecuador were preceded by a sharp increase in DENV cases in Colombia and Venezuela. This would indicate that human migration in concordance with increased number of infections due to local outbreaks and epidemics may intensify regional arboviral spread.

Interestingly, although DENV2 introduction into Ecuador in 2011 suggested Venezuela as possible origin, the Ecuadorian samples from 2014 to 2015 did not belong to the same cluster as the genomes sampled in Venezuela in 2015. Instead, the Ecuadorian genomes were in a cluster with a different virus variant that existed in Venezuela in 2007. This indicates that the virus that circulated in Venezuela in 2015 was vastly divergent from the one circulating in Ecuador at the same time, and the two had evolved independently since 1993. This should make any future estimations of more recent direct virus spread between the countries easier. It is, however, important to note that the Venezuelan origin of dengue in Ecuador by full genome analysis could not be supported by the E gene analyses. E gene is substantially shorter than the full genome, and less phylogenetic signal could have contributed to these discrepancies. However, it is also possible that more genomes in the E gene dataset, including more recent genomes, helped to, more granularly, resolve some of the viral relationships. These differences in the MCC trees indicate that care should be taken when interpreting results based on few genomes and/or short genomic regions. They also highlight the importance of more detailed genomic surveillance for inference of viral origins.

Despite the differences in the origins of Ecuadorian viruses, all our analyses indicated that Venezuela and Colombia to this day remain contributors of dissemination of dengue across this region of South America. This dissemination started already in the early 1990s, and has resulted in introduction and re-introduction of DENV1 and DENV2 into several countries of both South and North America, including Colombia, Argentina, Nicaragua, Puerto Rico, Brazil and Ecuador, resulting in sustained DENV spread in these countries [38–40]. The potential of dengue export from Venezuela over long distances has also been previously observed [41]. It is unclear why these countries emerge as sources of dengue spread for the past three decades in this region of South America. However, it is important to highlight that the exodus of Venezuelans, although massively increased in 2014–2019, started already in the late 1990s following the Bolivarian Revolution. Whether this is what contributed to the observed dissemination pattern of dengue across the region over the years is unclear; however, our results highlight that dengue has been historically disseminated from here even before the current massive refugee and migrant crisis. It would thus not be surprising if this trend also continued and was observed in the near future. Especially when timed with larger outbreaks and epidemics, this kind of human migration may lead to an increase in the number of regional viral introductions, and emergence of these conditions should be carefully monitored for immediate control measures. Venezuela contributed with many genomes in these analyses, which could have affected our observations. However, many other countries had more available genomes than Venezuela (Mexico, Brazil, Nicaragua, USA), and our down sampling to adjust for this skew should have minimized any errors. Nevertheless, sampling and full genome analyses from more recent time points and from additional countries associated with the current mass migration in South America, such as Peru, would be beneficial to infer more granular viral spread, and the hot spots of dengue export and import in this region. As our study contains samples from 2014 to 2015, and the migration from Venezuela has increased drastically after this time period, more contemporary samples and analyses could also reveal any changes in the patterns of spread described in this study, and would inform on how and to what extent the population migration in South America continues to affect arboviral dispersal.

Our analyses of the CHIKV outbreak in Machala reveled that this arbovirus, like dengue, was introduced into the country in at least two separate occasions, in 2014 and later that same year. Unlike dengue, however, CHIKV had been introduced from the Caribbean. CHIKV genomes from Colombia showed no correlation to the genomes from Ecuador, and were estimated introduced into this country from Martinique. Although CHIKV is spreading in Venezuela, no full genomes were available at the time of this study, making it impossible to infer the contribution of this country to the spread of CHIKV in the region [42]. Previous E gene analyses from Venezuela in 2014 did not provide enough resolution to determine the exact viral relationships, but did indicate a close connection to the CHIKV genomes from the Caribbean [43]. Thus, these and our analyses suggest that first early introductions were separately imported into these countries from outside of South America, and at least until 2015, there was no direct connection of CHIKV strains between these countries. However, more recent findings suggest that CHIKV infections are found close to the country borders, suggesting that CHIKV might have started following the patterns of dengue spread in this region [44]. Indeed, dengue, like CHIKV, was introduced into South America mainly through the Caribbean [40, 45, 46]. Given that these two pathogens share the common vector, *Ae. aegypti* in South America, the possibility of direct CHIKV dissemination between these countries of South America is real.

## Conclusion

In conclusion, we observe several introductions of arboviruses into Ecuador originating from different countries highlighting that Ecuador is vulnerable to arbovirus import from many geographic locations. DENV1 and DENV2 introductions, estimated to have originated in Venezuela and/or Colombia, were subsequent to a recent influx increase of Venezuelan and Colombian citizens into Ecuador, as well as to increases of dengue cases in these countries. However, Venezuela has historically played a major role in dengue dissemination in this area of the world, suggesting that paths and networks of viral spread may already be well established. Through these routes, regional virus dissemination becomes more likely during the occurrence of outbreaks and epidemics. This highlights the need of strengthening infectious disease surveillance along migration routes and improving access to healthcare for the vulnerable populations. It remains to be seen whether the recently introduced CHIKV, which as of 2015 was not directly spreading between the countries in this region, has started following these paths across the South American continent.

## Methods

### Data

De-identified samples were provided by *State University of New York (*SUNY) Upstate Medical University from an arbovirus surveillance study (January 2014 to December 2015) in the city of Machala, Ecuador (study design described previously) [6]. Briefly, subjects (> 6 months age) who were clinically diagnosed with dengue fever at MoH (Ministry of Health) clinic sentinel sites were eligible for participation in this study (index case). Subjects completed an informed consent or assent, as applicable, and research staff collected a blood specimen. Field teams visited the homes of index cases and recruited household members into the study, as well as household members from 4 homes located within 200 m of the index case home. Blood samples were tested at SUNY Upstate Medical University using qualitative real-time reverse transcriptase RT-PCR assays for DENV1–4, CHIKV, and ZIKV (diagnostic protocol described previously) [6]. RNA extracted from samples that were DENV and CHIKV positive by RT-PCR were sent to WRAIR, Viral Diseases Branch, for full-length sequencing. Samples from 2014 have been sequenced in a previous publication [6]. Samples from 2015 were sequenced in this study.

Migration data for Ecuador were obtained from INEC [33]. These data are from the Ecuadorian Registry of International Entries and Exits; we used the number of entries by country of nationality for the years 1997–2017. Maps were produced using GADM shapefiles, in ArcGIS v 10.6.1 [47, 48]. Dengue case count data was retrieved from Pan American Health Organization (PAHO) [34].

### Sequencing

Samples were extracted using QIAamp Viral RNA Mini QIACube Kit on QIACube (QIAGEN, Germantown, MD, USA). All sequencing was performed at the Walter Reed Army Institute of Research, Viral Diseases Branch. Here, extracted RNA was reverse transcribed and amplified utilizing serotype-specific DENV (DENV1 or DENV2) primers (Additional file 7-8) or CHIKV specific primers (Additional file 9). Two approaches of generating amplicons were performed, conventional PCR and integrated fluidic circuits (IFC) on the Access Array (Fluidigm, Palo Alto, CA). Up to 14 primer pairs were used for the conventional PCR and 48 primer pairs were used for IFC Access Array approach with both DENV-1 and DENV-2. For CHIKV, 24 primer pairs were used for both approaches. For the conventional PCR approach, the amplification was performed using Taq polymerase (ThermoFisher, Waltham, MA). The amplification via IFC was conducted with SSIII/HiFi Platinum Taq (Fluidigm, Palo Alto, CA). The reaction conditions for both approaches were 50 °C for 30 min and 94 °C for 2 min, followed by 35 cycles of 94 °C (30 s), 55 °C (30 s), and 68 °C (2 min), and a hold at 68 °C for 7 min prior to cooling down to 4 °C. Nextera XT libraries (Illumina, San Diego, CA) was utilized for library preparation prior to validation using Qubit (ThermoFisher, Waltham, MA) and TapeStation (Agilent, Santa Clara, CA). The libraries were normalized and pooled with equal molar ratio and the sequencing was conducted on the MiSeq reagent v.3600 cycles (Illumina, San Diego, CA).

### Genomes and alignments

Construction of dengue and chikungunya consensus genomes was done using ngs_mapper v1.2.4 in-house developed pipeline [49]. The consensus genomes were submitted to GenBank under accession numbers MN449007-MN449016 for DENV1, MN462632-MN462637 for DENV2 and MN462638-MN462662 for CHIKV. DENV1 genomes from Ecuador sequenced in this study were aligned to five previously published DENV1 genomes from Ecuador [6] using MEGAv7 [50], and to a set of full genome DENV1 reference genomes representing all genotypes, obtained from *National Center for Biotechnology Information’s* (NCBI’s) curated Virus Variation database [51], for genotype determination of the new Ecuadorian viruses. This DENV1 reference alignment was subsampled to represent genotype V and the American sub-lineage. The genomes were curated in TempEst [52] through linear regression of root to tip neighbor joining (NJ) tree distances given genome sampling times. Genomes with too much or too little divergence as would be expected based on their root to tip distance and collection date were considered as outliers and removed from the dataset. In addition, all genomes without collection location or date, or with long stretches of Ns, were removed from the alignment. A set of full genome DENV2 reference sequences was obtained following the same criteria as for DENV1, aligned to the new DENV2 sequenced genomes from Ecuador as well as to the previously published Ecuadorian genomes, and subsampled to the Asian American genotype [6]. The final DENV1 alignment consisted of 471 genomes, and DENV2 alignment of 655 genomes. In addition, all E gene sequences of DENV1 and 2 were downloaded from the Virus Pathogen Resource (ViPR) curated database [53], in order to increase the number of analyzed samples. The alignments were built following the same criteria for DENV1 and 2 full genomes, resulting in 1161 E genes of DENV1 and 1113 E genes of DENV2. All available CHIKV full genomes were downloaded from the ViPR curated database [53] and aligned to the newly sequenced genomes from Ecuador. A Neighbor Joining (NJ) tree was constructed to determine the lineage of the Ecuadorian genomes. Following curation in TempEst and removal of outlier genomes, a full genome alignment of the Asian lineage was constructed using all CHIKV sequences except genomes without collection location or date, or with long stretches of Ns (*N* = 352).

### Phylogenetic analyses

The best-fit models of evolution for DENV1, DENV2 and CHIKV datasets were determined using jModelTest v2.1.7 and chosen based on Bayesian Information Criterion (BIC) [54]. Maximum Likelihood (ML) phylogenetic trees for each of the DENV1, DENV2 and CHIKV datasets were inferred using Phyml v 4.9.1 [55] using the GTR + I + Γ (General Time Reversible + Invariable sites + Gamma distribution) model of evolution. Node confidence values were determined by aLRT (approximate Likelihood Ratio Test).

DENV1, DENV2 and CHIKV reference alignments were further down sampled for Bayesian tree reconstructions, such that all identical genomes from the same time and location were removed. Because of potential genome sampling skew, references were also down sampled for each DENV serotype, such that no country contributed with more than 40 genomes for the full genome alignments. This resulted in a DENV1 full genome BEAST dataset of 299 genomes (BEASTFGD1.large) and a DENV2 full genome BEAST dataset of 314 genomes (BEASTFGD2.large). Additionally, two smaller full genome BEAST datasets for DENV1 and DENV2 were built (BEASTFGD1.small and BEASTFGD2.small), with 103 and 117 genomes, respectively, in order to test the impact of missing data on phylogeographic reconstructions. For DENV1 and DENV2 E gene BEAST alignments, the genomes were downsampled such that no country contributed with more than 65 genes (*N* = 515 for DENV1 and *N* = 505 for DENV2). CHIKV full genome alignment for BEAST analyses consisted of 103 genomes.

BEAST [56] was ran for 600 million generations and sampling every 60,000 for BEASTFGD1.small and BEASTFGD2.small datasets, and 500 million generations and sampling every 50,000 for CHIKV. Bayesian Skyline coalescent prior was used, as was relaxed lognormal clock, geographic location discrete traits, a burn-in of 10% and Effective Sample Size (ESS) of a minimum of 200. For BEASTFGD1.large and BEASTFGD2.large each, a combination of three parallel BEAST runs was combined to a total of 890.34 and 1198.32 million generations, respectively, and sampling every 60,000 states. All three runs for each of the serotypes had overlapping traces, statistics and population distributions. For DENV1 E gene analyses, two parallel BEAST runs had overlapping traces, statistics and population distributions. Thus, a combination of those two parallel BEAST runs was used, with a total of 640.62 million generations and sampling every 60,000 states. For DENV2 E gene BEAST analyses, a combination of three parallel BEAST runs converged after 1208.82 million generations and sampling every 60,000 states.

## Supplementary information

**Additional file 1.** Arboviral genomes sequenced in this study from Machala, Ecuador.

**Additional file 2.** DENV1 full genome MCC tree on small dataset, BEASTFGD1.small. Taxa from Ecuador are color coded in green. Location origins are colored in the tree according to the legend. Times of the most recent common ancestors discussed in the text are noted next to the respective ancestor nodes.

**Additional file 3.** TMRCA and location probabilities of viral introductions in large and small datasets.

**Additional file 4.** DENV1 E gene MCC tree. Location origins are colored in the tree according to the legend. Times of the most recent common ancestors discussed in the text are noted next to the respective ancestor nodes.

**Additional file 5.** DENV2 full genome MCC tree on small dataset, BEASTFGD2.small. Taxa from Ecuador are color coded in green. Location origins are colored in the tree according to the legend. Times of the most recent common ancestors discussed in the text are noted next to the respective ancestor nodes.

**Additional file 6.** DENV2 E gene MCC tree. Location origins are colored in the tree according to the legend. Times of the most recent common ancestors discussed in the text are noted next to the respective ancestor nodes.

**Additional file 7.** DENV1 and DENV2 Specific Primer Pairs Used for Conventional PCR.

**Additional file 8.** DENV1 and DENV2 Specific Primer Pairs Used on the Integrated Fluidic Circuits of the Access Array (Fluidigm).

**Additional file 9.** CHIKV Specific Primer Pairs Used on the Integrated Fluidic Circuits of the Access Array (Fluidigm) and Conventional PCR.

## Data Availability

The novel genomes supporting the conclusions of this article have been submitted to GenBank under accession numbers: MN449007-MN449016 for DENV1, MN462632-MN462637 for DENV2 and MN462638-MN462662 for CHIKV.

## Abbreviations

Ae. aegypti: *Aedes aegypti*
Ae. albopictus: *Aedes albopictus*
aLRT: approximate Likelihood Ratio Test
BEAST: Bayesian Evolutionary Analysis by Sampling Trees
BIC: Bayesian Information Criterion
CHIKV: Chikungunya virus
DENV: Dengue virus
ESS: Effective Sample Size
GTR + I + Γ: General Time Reversible + Invariable sites + Gamma distribution
IFC: Integrated Fluidic Circuits
INEC: National Institute for Statistics and Census in Ecuador
MCC: Maximum clade credibility
ML: Maximum likelihood
MoH: Ministry of Health
MRCA: Most recent common ancestor
NCBI: National Center for Biotechnology Information
NJ: Neighbor joining
PAHO: Pan American Health Organization
PCR: Polymerase chain reaction
RNA: Ribonucleic acid
RT-PCR: Reverse transcription polymerase chain reaction
SUNY: State University of New York
TMRCA: Time of the most recent common ancestor
ViPR: Virus Pathogen Resource
ZIKV: Zika virus
