# Supplementary material for: The origins of dengue and chikungunya viruses in Ecuador following increased migration from Venezuela and Colombia
Source: BMC Evol Biol. 2020 Feb 19;20:31. doi: 10.1186/s12862-020-1596-8 (PMC7031975; doi:10.1186/s12862-020-1596-8)
Supplement: Supplementary file 1 — Additional file 1. Arboviral genomes sequenced in this study from Machala, Ecuador. [file 12862_2020_1596_MOESM1_ESM.docx]

Table S1. Genomes sequenced in this study from Machala, Ecuador, 2014-2015

| **Sequence name** | **Virus** | **Sampling date** | **Genotype/Lineage** |
| --- | --- | --- | --- |
| D1/Ecuador/00219-P_12902/2015 | DENV1 | 8/4/2015 | V |
| D1/Ecuador/00222-P_12903/2015 | DENV1 | 16/04/2015 | V |
| D1/Ecuador/00225-P_12904/2015 | DENV1 | 20/04/2015 | V |
| D1/Ecuador/00229-P_12905/2015 | DENV1 | 22/04/2015 | V |
| D1/Ecuador/00239-P_12906/2015 | DENV1 | 7/5/2015 | V |
| D1/Ecuador/00248-P_12907/2015 | DENV1 | 12/5/2015 | V |
| D1/Ecuador/00257-P_12908/2015 | DENV1 | 20/05/2015 | V |
| D1/Ecuador/00274-P_12909/2015 | DENV1 | 10/6/2015 | V |
| D1/Ecuador/00306-P_12910/2015 | DENV1 | 30/07/2015 | V |
| D2/Ecuador/00221-P_12912/2015 | DENV2 | 10/4/2015 | AmericanAsian |
| D2/Ecuador/00234-P_12913/2015 | DENV2 | 28/04/2015 | AmericanAsian |
| D2/Ecuador/00240-P_12914/2015 | DENV2 | 7/5/2015 | AmericanAsian |
| D2/Ecuador/00255-P_12915/2015 | DENV2 | 19/05/2015 | AmericanAsian |
| D2/Ecuador/00265-P_12916/2015 | DENV2 | 27/05/2015 | AmericanAsian |
| D2/Ecuador/00280-P12917/2015 | DENV2 | 23/06/2015 | AmericanAsian |
| CHIKV/Ecuador/00212-S/2015 | CHIKV | 24/03/2015 | Asian |
| CHIKV/Ecuador/00218-P/2015 | CHIKV | 08/04/2015 | Asian |
| CHIKV/Ecuador/00235-P/2015 | CHIKV | 04/05/2015 | Asian |
| CHIKV/Ecuador/00236-P/2015 | CHIKV | 05/05/2015 | Asian |
| CHIKV/Ecuador/00246-P/2015 | CHIKV | 11/05/2015 | Asian |
| CHIKV/Ecuador/00247-P/2015 | CHIKV | 11/05/2015 | Asian |
| CHIKV/Ecuador/00252-P/2015 | CHIKV | 15/05/2015 | Asian |
| CHIKV/Ecuador/00261-P/2015 | CHIKV | 26/05/2015 | Asian |
| CHIKV/Ecuador/00264-P/2015 | CHIKV | 27/05/2015 | Asian |
| CHIKV/Ecuador/00267-P/2015 | CHIKV | 28/05/2015 | Asian |
| CHIKV/Ecuador/00268-P/2015 | CHIKV | 28/05/2015 | Asian |
| CHIKV/Ecuador/00272-P/2015 | CHIKV | 02/06/2015 | Asian |
| CHIKV/Ecuador/00278-P/2015 | CHIKV | 16/06/2015 | Asian |
| CHIKV/Ecuador/00283-P/2015 | CHIKV | 24/06/2015 | Asian |
| CHIKV/Ecuador/00284-P/2015 | CHIKV | 30/06/2015 | Asian |
| CHIKV/Ecuador/00285-P/2015 | CHIKV | 30/06/2015 | Asian |
| CHIKV/Ecuador/00287-P/2015 | CHIKV | 02/07/2015 | Asian |
| CHIKV/Ecuador/00290-P/2015 | CHIKV | 14/07/2015 | Asian |
| CHIKV/Ecuador/00294-P/2015 | CHIKV | 23/07/2015 | Asian |
| CHIKV/Ecuador/00304-P/2015 | CHIKV | 29/07/2015 | Asian |
| CHIKV/Ecuador/00308-P/2015 | CHIKV | 04/08/2015 | Asian |
| CHIKV/Ecuador/00311-P/2015 | CHIKV | 12/08/2015 | Asian |
| CHIKV/Ecuador/00312-P/2015 | CHIKV | 14/08/2015 | Asian |
| CHIKV/Ecuador/00313-P/2015 | CHIKV | 14/08/2015 | Asian |
| CHIKV/Ecuador/00316-P/2015 | CHIKV | 27/08/2015 | Asian |
