# Supplementary material for: The origins of dengue and chikungunya viruses in Ecuador following increased migration from Venezuela and Colombia
Source: BMC Evol Biol. 2020 Feb 19;20:31. doi: 10.1186/s12862-020-1596-8 (PMC7031975; doi:10.1186/s12862-020-1596-8)
Supplement: Supplementary file 2 — Additional file 2. DENV1 full genome MCC tree on small dataset, BEASTFGD1.small. Taxa from Ecuador are color coded in green. Location origins are colored in the tree according to the legend. Times of the most recent common ancestors discussed in the text are noted next to the respective ancestor nodes. [file 12862_2020_1596_MOESM2_ESM.pdf]

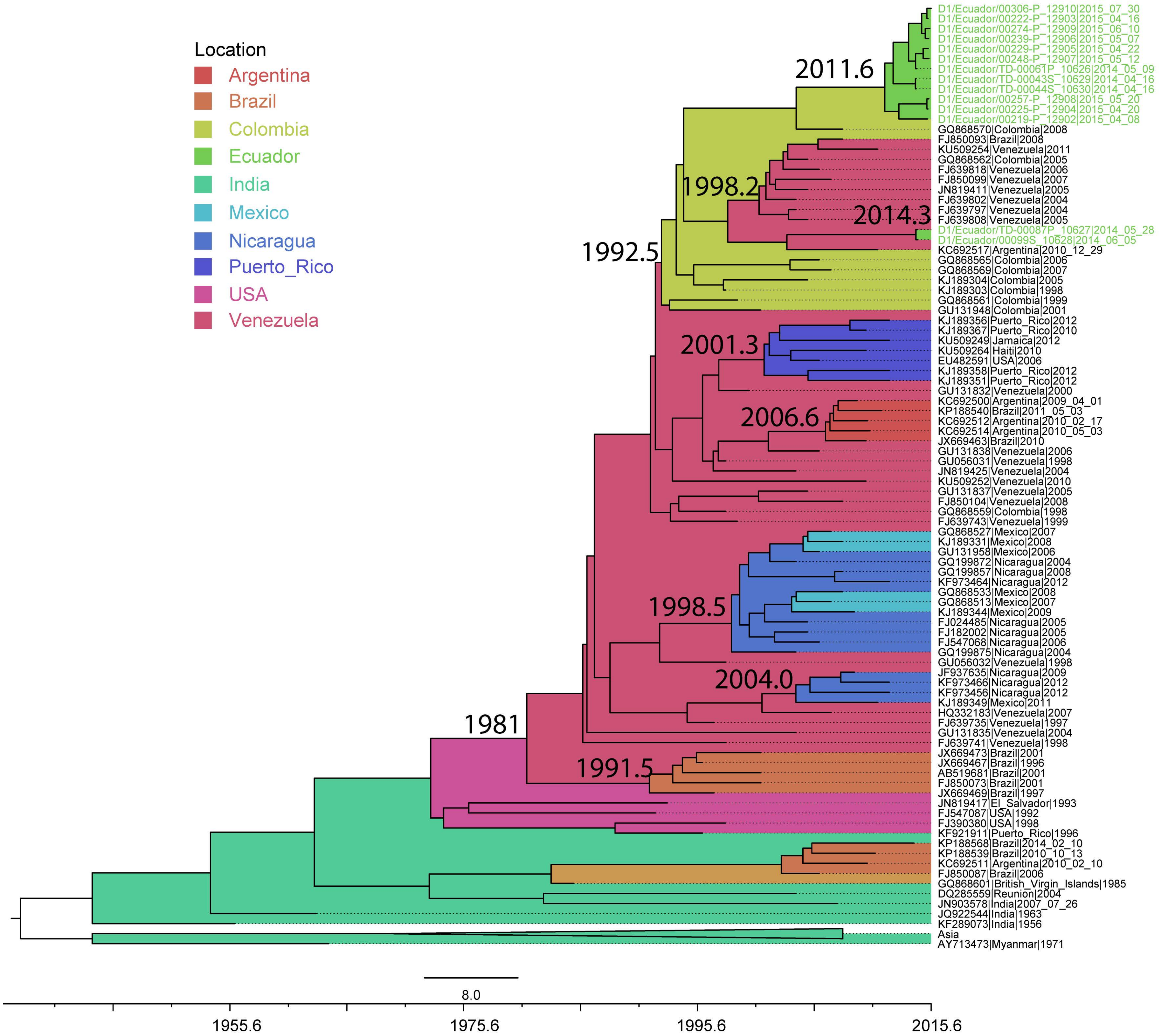

Location
Argentina
Brazil
Colombia
Ecuador
India
Mexico
Nicaragua
Puerto_Rico
USA
Venezuela
2011.6
1998.2
2014.3
1992.5
2001.3
2006.6
1998.5
2004.0
1981
1991.5
D1/Ecuador/00306-P_12910|2015_07_30
D1/Ecuador/00222-P_12903|2015_04_16
D1/Ecuador/00274-P_12909|2015_06_10
D1/Ecuador/00239-P_12906|2015_05_07
D1/Ecuador/00229-P_12905|2015_04_22
D1/Ecuador/00248-P_12907|2015_05_12
D1/Ecuador/TD-00061P_10626|2014_05_09
D1/Ecuador/TD-00043S_10629|2014_04_16
D1/Ecuador/TD-00044S_10630|2014_04_16
D1/Ecuador/00257-P_12908|2015_05_20
D1/Ecuador/00225-P_12904|2015_04_20
D1/Ecuador/00219-P_12902|2015_04_08
GQ868570|Colombia|2008
FJ850093|Brazil|2008
KU509254|Venezuela|2011
GQ868562|Colombia|2005
FJ639818|Venezuela|2006
FJ850099|Venezuela|2007
JN819411|Venezuela|2005
FJ639802|Venezuela|2004
FJ639797|Venezuela|2004
FJ639808|Venezuela|2005
D1/Ecuador/TD-00087P_10627|2014_05_28
D1/Ecuador/00099S_10628|2014_06_05
KC692517|Argentina|2010_12_29
GQ868565|Colombia|2006
GQ868569|Colombia|2007
KJ189304|Colombia|2005
KJ189303|Colombia|1998
GQ868561|Colombia|1999
GU131948|Colombia|2001
KJ189356|Puerto_Rico|2012
KJ189367|Puerto_Rico|2010
KU509249|Jamaica|2012
KU509264|Haiti|2010
EU482591|USA|2006
KJ189358|Puerto_Rico|2012
KJ189351|Puerto_Rico|2012
GU131832|Venezuela|2000
KC692500|Argentina|2009_04_01
KP188540|Brazil|2011_05_03
KC692512|Argentina|2010_02_17
KC692514|Argentina|2010_05_03
JX669463|Brazil|2010
GU131838|Venezuela|2006
GU056031|Venezuela|1998
JN819425|Venezuela|2004
KU509252|Venezuela|2010
GU131837|Venezuela|2005
FJ850104|Venezuela|2008
GQ868559|Colombia|1998
FJ639743|Venezuela|1999
GQ868527|Mexico|2007
KJ189331|Mexico|2008
GU131958|Mexico|2006
GQ199872|Nicaragua|2004
GQ199857|Nicaragua|2008
KF973464|Nicaragua|2012
GQ868533|Mexico|2008
GQ868513|Mexico|2007
KJ189344|Mexico|2009
FJ024485|Nicaragua|2005
FJ182002|Nicaragua|2005
FJ547068|Nicaragua|2006
GQ199875|Nicaragua|2004
GU056032|Venezuela|1998
JF937635|Nicaragua|2009
KF973466|Nicaragua|2012
KF973456|Nicaragua|2012
KJ189349|Mexico|2011
HQ332183|Venezuela|2007
FJ639735|Venezuela|1997
GU131835|Venezuela|2004
FJ639741|Venezuela|1998
JX669473|Brazil|2001
JX669467|Brazil|1996
AB519681|Brazil|2001
FJ850073|Brazil|2001
JX669469|Brazil|1997
JN819417|El_Salvador|1993
FJ547087|USA|1992
FJ390380|USA|1998
KF921911|Puerto_Rico|1996
KP188568|Brazil|2014_02_10
KP188539|Brazil|2010_10_13
KC692511|Argentina|2010_02_10
FJ850087|Brazil|2006
GQ868601|British_Virgin_Islands|1985
DQ285559|Reunion|2004
JN903578|India|2007_07_26
JQ922544|India|1963
KF289073|India|1956
Asia
AY713473|Myanmar|1971
8.0
1955.6
1975.6
1995.6
2015.6
