# Supplementary material for: The origins of dengue and chikungunya viruses in Ecuador following increased migration from Venezuela and Colombia
Source: BMC Evol Biol. 2020 Feb 19;20:31. doi: 10.1186/s12862-020-1596-8 (PMC7031975; doi:10.1186/s12862-020-1596-8)
Supplement: Supplementary file 3 — Additional file 3. TMRCA and location probabilities of viral introductions in large and small datasets. [file 12862_2020_1596_MOESM3_ESM.docx]

Table S2. Time of the most recent common ancestor (TMRCA), its highest posterior density (HPD), and origin (donor) probabilities of viral introductions into the countries of S. America in the large and small dataset analyses.

|  |  | **Large dataset** | | **Small dataset** | |
| --- | --- | --- | --- | --- | --- |
| Serotype | Donor-Recipient country | TMRCA recipient (95% HPD) | Donor probability | TMRCA recipient (95% HPD) | Donor probability |
| 1 | Venezuela - Argentina | 2006.8 (2006-2007.4) | 1 | 2006.6 (2004.7-2008) | 1 |
|  | Venezuela - Brazil | *ND* | *ND* | 1991.5 (1988.6-1993.6) | 0.62 |
|  | Venezuela - Colombia | 1993.2 (1990.4-1996.2) | 0.3 | 1992.5 (1990.7-1994.3) | 0.97 |
|  | Venezuela - Colombia | 1995.4 (1993.7-1996.9) | 0.83 | *ND* | *ND* |
|  | Venezuela - Colombia | 1995.9 (1994.0 -1997.3) | 1 | *ND* | *ND* |
|  | Venezuela - Ecuador | 2013.9 (2013.5-2014.1) | 1 | 2014.3 (2014-2014.4) | 0.33 |
|  | *Venezuela - Ecuador | 2011.1 (2009.5-2012.3) | 1 | *ND* | *ND* |
|  | *Colombia - Ecuador | *ND* | *ND* | 2011.6 (2010.2-2012.8) | 0.86 |
|  | Venezuela - Nicaragua | 1998.2 (1996.5-1999.9) | 1 | 1998.5 (1996.3-2000.6) | 0.99 |
|  | Venezuela - Nicaragua | 2004.4 (2002.2-2006.1) | 1 | 2004 (2001.6-2006.3) | 0.98 |
|  | Venezuela - Puerto Rico | 2001.4 (1998.7-2003.6) | 1 | 2001.3 (1999.3-2003.5) | 1 |
|  | Colombia - Venezuela | *ND* | *ND* | 1998.2 (1995.8-2000.5) | 0.65 |
|  | Argentina - Brazil | 2008.8 (2008.1-2009.4) | 1 | *ND* | *ND* |
|  | Argentina - Brazil | 2008.2 (2007.3-2009.1) | 1 | *ND* | *ND* |
|  | Brazil - Venezuela | 1985.2 (1981.5-1988.2) | 1 | *ND* | *ND* |
| 2 | Venezuela - Ecuador | 2011.3 (2010.5-2012.0) | 0.99 | 2010.8 (2009.9-2011.7) | 0.67 |
|  | Venezuela - Colombia | 2004.1 (2003.6-2004.5) | 0.98 | 2002.3 (2001.6-2002.9) | 0.75 |
|  | Venezuela - Colombia | 2002.7 (2001.8-2003.5) | 0.99 | 2002.6 (2001.9-2003.3) | 0.76 |
|  | Venezuela - Nicaragua | 1996.8 (1995.9-1997.5) | 0.80 | 1997 (1996.2-1997.7) | 0.61 |
|  | Venezuela - Brazil | 1989.1 (1988.4-1989.6) | 0.54 | 1991 (1989.4-1992.6) | 0.47 |
|  | Brazil - Peru | 2010.2 (2009.9-2010.4) | 0.98 | 2010.3 (2010.1-2010.7) | 0.99 |
|  | Brazil - Peru | 2006.4 (2005.5-2007.1) | 1 | *ND* | *ND* |
|  | Brazil - Peru | 2001.8 (2001.4-2002.1) | 1 | 2001.8 (2001.5-2002) | 0.98 |
| * Ecuador introduction discrepancy between the trees  ND-not detected | | |  |  |  |
