# Supplementary figures and images for: The origins of dengue and chikungunya viruses in Ecuador following increased migration from Venezuela and Colombia
Source: BMC Evol Biol. 2020 Feb 19;20:31. doi: 10.1186/s12862-020-1596-8 (PMC7031975; doi:10.1186/s12862-020-1596-8)

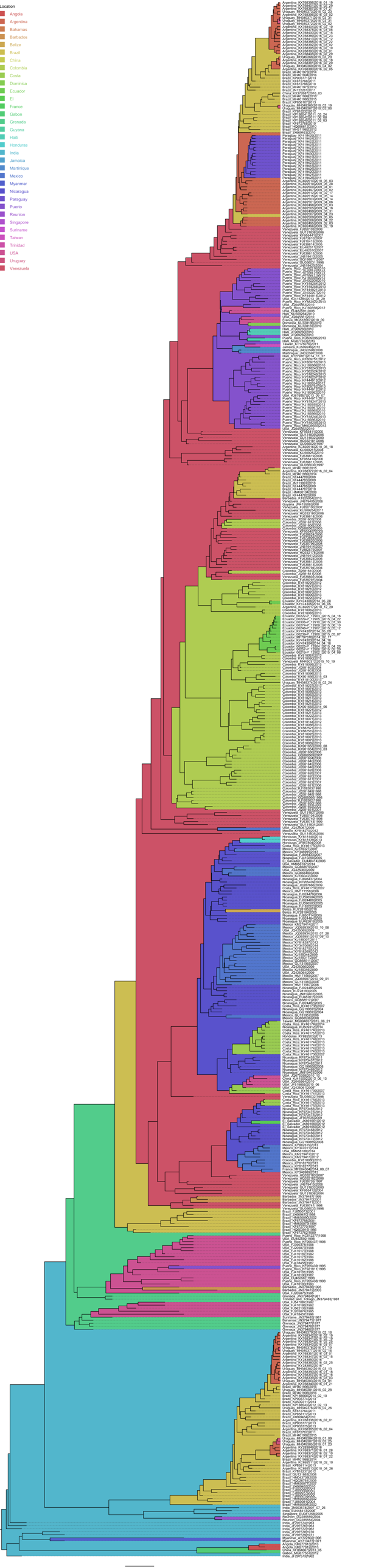

Supplement: Supplementary file 4 — Additional file 4. DENV1 E gene MCC tree. Location origins are colored in the tree according to the legend. Times of the most recent common ancestors discussed in the text are noted next to the respective ancestor nodes. [file 12862_2020_1596_MOESM4_ESM.pdf]

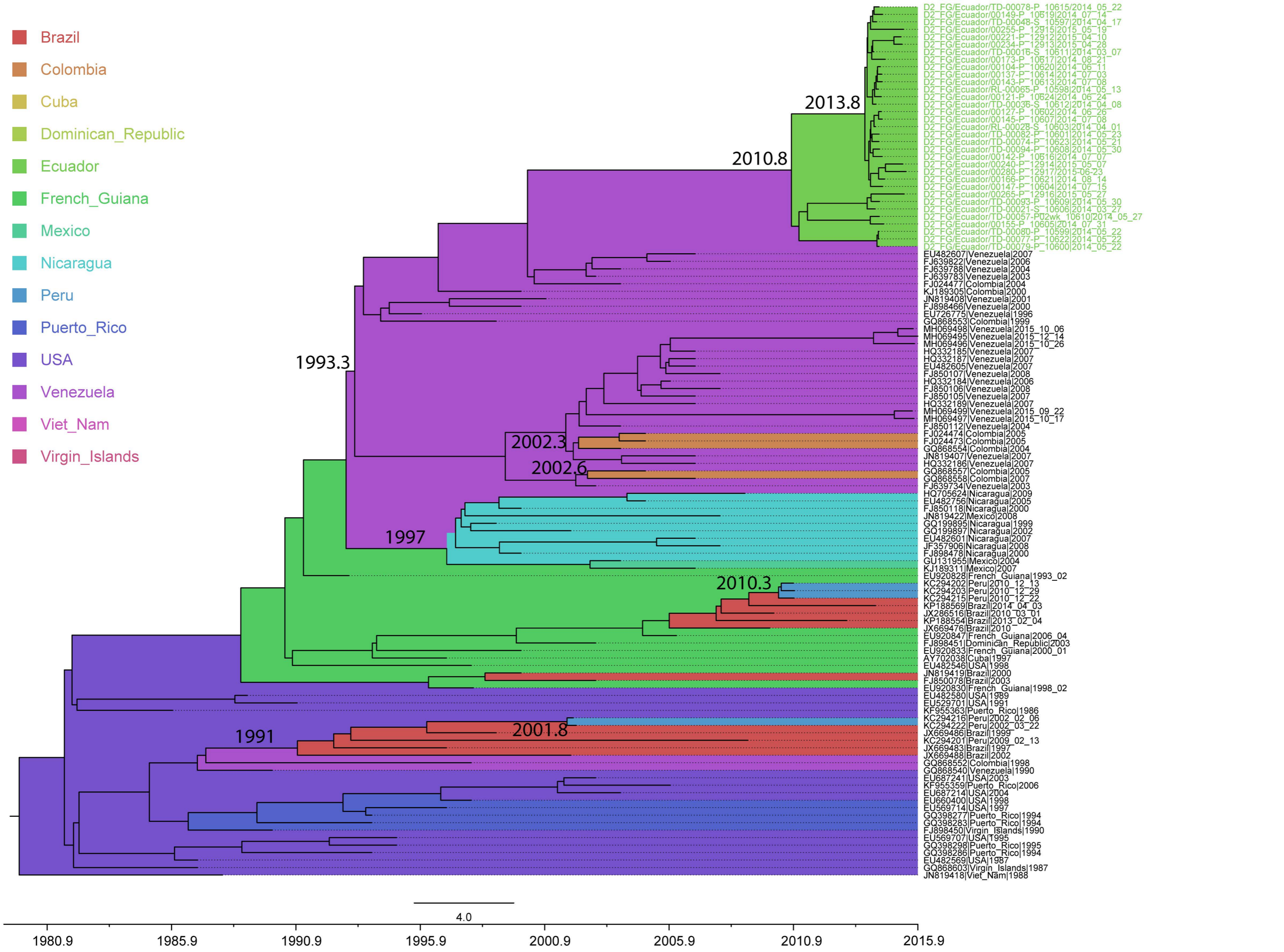

Supplement: Supplementary file 5 — Additional file 5. DENV2 full genome MCC tree on small dataset, BEASTFGD2.small. Taxa from Ecuador are color coded in green. Location origins are colored in the tree according to the legend. Times of the most recent common ancestors discussed in the text are noted next to the respective ancestor nodes. [file 12862_2020_1596_MOESM5_ESM.pdf]

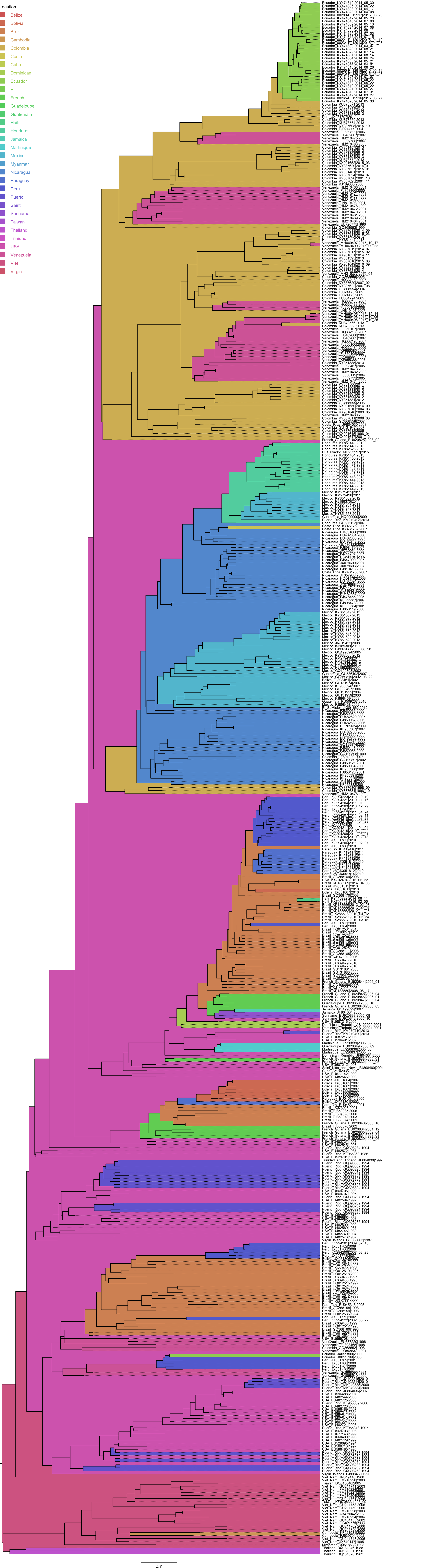

Supplement: Supplementary file 6 — Additional file 6. DENV2 E gene MCC tree. Location origins are colored in the tree according to the legend. Times of the most recent common ancestors discussed in the text are noted next to the respective ancestor nodes. [file 12862_2020_1596_MOESM6_ESM.pdf]
