# Supplementary material for: The origins of dengue and chikungunya viruses in Ecuador following increased migration from Venezuela and Colombia
Source: BMC Evol Biol. 2020 Feb 19;20:31. doi: 10.1186/s12862-020-1596-8 (PMC7031975; doi:10.1186/s12862-020-1596-8)
Supplement: Supplementary file 7 — Additional file 7. DENV1 and DENV2 Specific Primer Pairs Used for Conventional PCR. [file 12862_2020_1596_MOESM7_ESM.docx]

Table S2: DENV1 and DENV2 Specific Primer Pairs Used for Conventional PCR

| **Primer Pairs** | **Forward Primer** | **Sequences** | **Reverse Primer** | **Sequences** | **Amplicon**  **Size (bp)** | **Virus** |
| --- | --- | --- | --- | --- | --- | --- |
| 1 | 5’D1F1 | AGTTGTTAGTCTACGTGG | 3’D1R1098 | ACGGCAGGGTTTGTGACTT | 1098 | DENV1 |
| 2 | 5’D1F970 | GGAAGGACTGTCAGGAGCAA | 3’D1R2009 | TGACTATGGGGTTGGCTGTT | 1040 | DENV1 |
| 3 | 5’D1F1839 | GCACAGGCTCATTCAAGCTA | 3’D1R2882 | CTTCCCAAATGTTCCATGCT | 1044 | DENV1 |
| 4 | 5’D1F2769 | GCTGGGGAAAGGCTAAAATC | 3’D1R3716 | AAGTGGCCATCAGAGCTAGG | 948 | DENV1 |
| 5 | 5’D1F3571 | GATGACTGGAACACTGGCTGT | 3’D1R4562 | TTTCCACTTCTGGAGGGCTA | 992 | DENV1 |
| 6 | 5’D1F4397 | GAGAGAGATGACACGCTAACCA | 3’D1R5419 | CACTCGGGTTGARATGTA | 1023 | DENV1 |
| 7 | 5’D1F4026 | ACAACATGGCTTCCGGTG | 3’D1R5347 | AACTCTCACGGGAGACAGGA | 1322 | DENV1 |
| 8 | 5’D1F5226 | TCAAGGGAATGCCAATAAGG | 3’D1R6305 | AGCGGGGTCGTAGTTTCTTT | 1080 | DENV1 |
| 9 | 5’D1F6199 | CAGAAGATGGTGCTTTGACG | 3’D1R7140 | GGATTCACCTGGGAATAGCA | 942 | DENV1 |
| 10 | 5’D1F6978 | CCATGATGAGGCACACAATC | 3’D1R8036 | TTGGGTTTGGAGAGGACTCA | 1059 | DENV1 |
| 11 | 5’D1F7707 | ACAACCAAACATGCAGTGTC | 3’D1R8974 | TGCCTTTCCGAACTCTCCTA | 1268 | DENV1 |
| 12 | 5’D1F7894 | ACCTGGACATGAGGAACCAA | 3’D1R8974 | TGCCTTTCCGAACTCTCCTA | 1081 | DENV1 |
| 13 | 5’D1F8884 | GAGGGAGCTTCACAAACAGG | 3’D1R9924 | ACTGGAACGGCTGAACAGAT | 1041 | DENV1 |
| 14 | 5’D1F9727 | GGATGGGAGGGAAATAGTGG | 3’D1R10735 | AGAACCTGTTGATTCAAC | 1009 | DENV1 |
| 1 | 5’D2F1 | AGTAGTTAGTCTACGTGGAC | 3’D2R1032 | CGTCACACAGCTTCCATGTT | 1032 | DENV2 |
| 2 | 5’D2F871 | GGGACGACACATTTCCAGAG | 3’D2R1929 | AGAGCCGTCCCCTTCATATT | 1059 | DENV2 |
| 3 | 5’D2F1784 | TCAAGTGCAGGCTGAGAATG | 3’D2R2801 | TCTGTGGAGAGCATTTTTGC | 1018 | DENV2 |
| 4 | 5’D2F2587 | GGGATCCGCTCAGTAACAAG | 3’D2R3694 | TCACGCCCATACCTATGTCA | 1108 | DENV2 |
| 5 | 5’D2F3526 | TTGTTCCTGGAGGAAATGCT | 3’D2R4592 | CTGTAGGCTCCGTCTTCCAG | 1067 | DENV2 |
| 6 | 5’D2F4488 | AGCATGGTACCTGTGGGAAG | 3’D2R5519 | ATGATTGGTGCATTGCTCTG | 1032 | DENV2 |
| 7 | 5’D2F5372 | ACGAAGCCCATTTCACAGAC | 3’D2R6454 | TGTCCAGTGCGTCTCTTGTC | 1083 | DENV2 |
| 8 | 5’D2F6264 | CTGGACAAAAGAAGGGGAAAG | 3’D2R7239 | CGCTGCTCTTTTCTGAGCTT | 976 | DENV2 |
| 9 | 5’D2F7028 | TAGCCAACCAAGCAACAGTG | 3’D2R8136 | TGAGGGCATATATGGGTTGAG | 1109 | DENV2 |
| 10 | 5’D2F8025 | AAATGGAAGCACTGCAAAGG | 3’D2R8966 | CCGAATTCCCCTAGCTTCTT | 942 | DENV2 |
| 11 | 5’D2F8792 | CCTTGGGAGCCATATTCACT | 3’D2R9773 | GGCTCTGCCAATCAGTTCAT | 982 | DENV2 |
| 12 | 5’D2F9543 | GGCCATCAGTGGAGATGATT | 3’D2R10723 | AGAACCTGTTGATTCAAC | 1181 | DENV2 |
