# Supplementary material for: The origins of dengue and chikungunya viruses in Ecuador following increased migration from Venezuela and Colombia
Source: BMC Evol Biol. 2020 Feb 19;20:31. doi: 10.1186/s12862-020-1596-8 (PMC7031975; doi:10.1186/s12862-020-1596-8)
Supplement: Supplementary file 8 — Additional file 8. DENV1 and DENV2 Specific Primer Pairs Used on the Integrated Fluidic Circuits of the Access Array (Fluidigm). [file 12862_2020_1596_MOESM8_ESM.docx]

Table S3. DENV1 and DENV2 Specific Primer Pairs Used on the Integrated Fluidic Circuits of the Access Array (Fluidigm)

| Primer Pair | Forward Primer | Sequence | Reverse Primer | Sequence | Amplicon Size (bp) | Virus |
| --- | --- | --- | --- | --- | --- | --- |
| 1 | 5’D1Ffirstb | AGTTGTTAGTCTACGTGG | 3’D1R1098 | ACGGCAGGGTTTGTGACTT | 1098 | DENV1 |
| 2 | 5’D1Ffirstb | AGTTGTTAGTCTACGTGG | 3’D1R645 | GCATTGCACCAACAATCAAC | 645 | DENV1 |
| 3 | 5’D1F386 | GACCATGCTCCTNATGCTG | 3’D1R1127b | GGTGGTGTTTGATATTTTAG | 742 | DENV1 |
| 4 | 5’D1F546 | TAGCGATGGATTTGGGAGAG | 3’D1R1599 | GAGGTCCAAGGCAGTGGTAA | 1054 | DENV1 |
| 5 | 5’D1F751b | GGATGTCCTCTGAAGGCGC | 3’D1R1703 | GTGCATTGCTCCTTCTTG | 953 | DENV1 |
| 6 | 5’D1F970 | GGAAGGACTGTCAGGAGCAA | 3’D1R2009 | TGACTATGGGGTTGGCTGTT | 1040 | DENV1 |
| 7 | 5’D1F1035 | CCAACATTGGACATTGAAC | 3’D1R2009 | TGACTATGGGGTTGGCTGTT | 975 | DENV1 |
| 8 | 5’D1F1458 | TGACCGACTACGGAGCTCTT | 3’D1R2419 | CGCCTGAACCATGACTCCTA | 962 | DENV1 |
| 9 | 5’D1F1545 | CACAAACAATGGTTTCTAG | 3’D1R2581b | GCTGATCGAATTCCACACAC | 1037 | DENV1 |
| 10 | 5’D1F1835 | ATGTGCACAGGCTCATTCAA | 3’D1R2625 | GTTCATTTGATATTTGCTTC | 791 | DENV1 |
| 11 | 5’D1F1835 | ATGTGCACAGGCTCATTCAA | 3’D1R2882 | CTTCCCAAATGTTCCATGCT | 1048 | DENV1 |
| 12 | 5’D1F2139 | GGAGCACGAAGGATGGC | 3’D1R2979 | TNCTGTCCTTGATGGCAG | 841 | DENV1 |
| 13 | 5’D1F2317 | TCTGCTGACATGGCTAGGATT | 3’D1R3411 | TACCAGCACCCATCTTCTCC | 1095 | DENV1 |
| 14 | 5’D1F2483b | CACTTGGACAGAGCAATA | 3’D1R3456 | GAGACCATTGANNTNACTA | 974 | DENV1 |
| 15 | 5’D1F2769 | GCTGGGGAAAGGCTAAAATC | 3’D1R3716 | AAGTGGCCATCAGAGCTAGG | 948 | DENV1 |
| 16 | 5’D1F2862 | TGGGAAGTTGAGGACTATG | 3’D1R3760 | TCTCTGGATGTTANTCTGC | 899 | DENV1 |
| 17 | 5’D1F3161 | GGAGGACCAATATCTCAGCA | 3’D1R4158 | GCCATGATCCCTTCATTGAG | 998 | DENV1 |
| 18 | 5’D1F3264 | GTGGATGAACATTGTGGAA | 3’D1R4226 | CATGCCTCCAGCTATTAGT | 963 | DENV1 |
| 19 | 5’D1F3571 | GATGACTGGAACACTGGCTGT | 3’D1R4562 | TTTCCACTTCTGGAGGGCTA | 992 | DENV1 |
| 20 | 5’D1F3604 | TGACATGGAATGATCTGAT | 3’D1R4603 | CTGCCCAACAGTCCTCT | 1000 | DENV1 |
| 21 | 5’D1F4012 | CTTATGCCTGTCCACGACCT | 3’D1R4951 | TCCTTCTCTGTTCACGATGG | 940 | DENV1 |
| 22 | 5’D1F4026 | ACAACATGGCTTCCGGTG | 3’D1R5347 | AACTCTCACGGGAGACAGGA | 1322 | DENV1 |
| 23 | 5’D1F4397 | GAGAGAGATGACACGCTAACCA | 3’D1R5419 | CACTCGGGTTGARATGTA | 1023 | DENV1 |
| 24 | 5’D1F4529 | AGCCCTCCAGAAGTGGAA | 3’D1R5419 | CACTCGGGTTGARATGTA | 891 | DENV1 |
| 25 | 5’D1F4783 | CTGGAACACGGGAGAAGAAG | 3’D1R5810 | TCAGACACCGTCTTGGGTCT | 1028 | DENV1 |
| 26 | 5’D1F4864 | AAGGNGAAGTTGGAGCCAT | 3’D1R5810 | TCAGACACCGTCTTGGGTCT | 947 | DENV1 |
| 27 | 5’D1F5226 | TCAAGGGAATGCCAATAAGG | 3’D1R6168 | CTTCTGAGGCAACTTTGTA | 943 | DENV1 |
| 28 | 5’D1F5348 | GATTATCATGGATGAAGCAC | 3’D1R6305 | AGCGGGGTCGTAGTTTCTTT | 958 | DENV1 |
| 29 | 5’D1F5673b | AACCTTTGACACNGAGTA | 3’D1R6667 | GGCTCCACACTGGCCATC | 995 | DENV1 |
| 30 | 5’D1F5675 | TTGAGCAGGAAAACCTTTGA | 3’D1R6667 | GGCTCCACACTGGCCATC | 993 | DENV1 |
| 31 | 5’D1F6117 | ATGAGAAGAGGNGATNTA | 3’D1R7076 | TCCATCTTCGATATTGGC | 960 | DENV1 |
| 32 | 5’D1F6199 | CAGAAGATGGTGCTTTGACG | 3’D1R7140 | GGATTCACCTGGGAATAGCA | 942 | DENV1 |
| 33 | 5’D1F6554 | ACTGGTGGAGTGACGCTG | 3’D1R7405 | GCCAGTGTGATGGATTC | 852 | DENV1 |
| 34 | 5’D1F6569 | ACTGGTGGAGTGACGCTGTT | 3’D1R7535 | CCAGACCTGCTCCTGCTAGA | 967 | DENV1 |
| 35 | 5’D1F6920b | TTCAGCCTGGACNCTCTAT | 3’D1R7819 | AGCCACCTCTTCCACAAC | 900 | DENV1 |
| 36 | 5’D1F6978 | CCATGATGAGGCACACAATC | 3’D1R8036 | TTGGGTTTGGAGAGGACTCA | 1059 | DENV1 |
| 37 | 5’D1F7284b | CCTGTGGTTTANGATGCAA | 3’D1R8237 | GTTTCCTGTNCCACATGA | 954 | DENV1 |
| 38 | 5’D1F7397 | TTGTGCGAATCCATCACACT | 3’D1R8491 | TCCATGATAGGCCCATGTTT | 1095 | DENV1 |
| 39 | 5’D1F7707 | ACAACCAAACATGCAGTGTC | 3’D1R8974 | TGCCTTTCCGAACTCTCCTA | 1268 | DENV1 |
| 40 | 5’D1F7894 | ACCTGGACATGAGGAACCAA | 3’D1R8974 | TGCCTTTCCGAACTCTCCTA | 1081 | DENV1 |
| 41 | 5’D1F8052 | CTAAAGATGGTGGAACCAT | 3’D1R9014b | ACTCTAGAAAGCGTGCTC | 963 | DENV1 |
| 42 | 5’D1F8370 | TGGAACCAGAGGTAGCCAAC | 3’D1R9432 | CAGACTCCATTTGTCTTAT | 1063 | DENV1 |
| 43 | 5’D1F8485b | AGGTNAAGCCATCAGGAT | 3’D1R9432 | CAGACTCCATTTGTCTTAT | 948 | DENV1 |
| 44 | 5’D1F8861 | GCACAGAGAGAGGGAGCT | 3’D1R9862 | CCTGTGGAANTACATCAGC | 1002 | DENV1 |
| 45 | 5’D1F8884 | GAGGGAGCTTCACAAACAGG | 3’D1R9924 | ACTGGAACGGCTGAACAGAT | 1041 | DENV1 |
| 46 | 5’D1F9326 | AATGGAACCGTGATGGATGT | 3’D1R10219 | CTCTTCATTGATGTCATGTA | 894 | DENV1 |
| 47 | 5’D1F9726 | ATAGTGGTGCCATGCCGC | 3’D1R10735b | AGAACCTGTTGATTCAAC | 1010 | DENV1 |
| 48 | 5’D1F9727 | GGATGGGAGGGAAATAGTGG | 3’D1R10735b | AGAACCTGTTGATTCAAC | 1009 | DENV1 |
| 1 | 5’D2F1 | AGTAGTTAGTCTACGTGGAC | 3'D2R1032 | CGTCACACAGCTTCCATGTT | 1032 | DENV2 |
| 2 | 5’D2F1 | AGTAGTTAGTCTACGTGGAC | 3'D2R604 | GAAGGGGACACTTGTACGTGA | 604 | DENV2 |
| 3 | 5'D2F12 | TACGTGGACCGACAAAGACA | 3’D2R940 | GCATTGTCATTGAAGGAG | 929 | DENV2 |
| 4 | 5'D2F448 | ACCACACGTAACGGAGAACC | 3'D2R1420 | TTTCCTTGCCATGTTTTCCT | 973 | DENV2 |
| 5 | 5’D2F460 | GGAGAACCACACATGATC | 3’D2R1264 | TTCCAAATAATCCACATC | 805 | DENV2 |
| 6 | 5'D2F871 | GGGACGACACATTTCCAGAG | 3'D2R1929 | AGAGCCGTCCCCTTCATATT | 1059 | DENV2 |
| 7 | 5’D2F798 | CCAGAGAATTGAAACTTGGA | 3’D2R1695 | TAAAACAACAACATCCTG | 898 | DENV2 |
| 8 | 5’D2F1200 | GAGGTTCGTCTGCAAACA | 3’D2R2099 | TTCAGTTGTCCCGGCTCT | 900 | DENV2 |
| 9 | 5'D2F1307 | ACATGGAAGGGAAAATCGTG | 3'D2R2369 | GACACAGACAGTGAGGTGCTG | 1063 | DENV2 |
| 10 | 5’D2F1729 | ACAGGAGCCACAGAAATCCA | 3’D2R2516 | GTATTGTTCTGTCCATGTGT | 788 | DENV2 |
| 11 | 5'D2F1784 | TCAAGTGCAGGCTGAGAATG | 3'D2R2801 | TCTGTGGAGAGCATTTTTGC | 1018 | DENV2 |
| 12 | 5’D2F2122 | AGTTCTATCGGCCAAATG | 3’D2R2830 | TTGTGTTGGGGCATTCTG | 709 | DENV2 |
| 13 | 5'D2F2204 | GATCCCTGGGAGGAGTGTTT | 3'D2R3226 | GCCATGGTCCTGCTATTTGT | 1023 | DENV2 |
| 14 | 5’D2F2410 | ATGGTGCAGGCCGATAGT | 3’D2R3276 | TCCATCGCAGAAATCAAAGT | 867 | DENV2 |
| 15 | 5'D2F2587 | GGGATCCGCTCAGTAACAAG | 3'D2R3694 | TCACGCCCATACCTATGTCA | 1108 | DENV2 |
| 16 | 5’D2F2815 | ACCTTTCTCATTGATGGC | 3’D2R3726 | AACTTTGAAGGCTGCTAG | 912 | DENV2 |
| 17 | 5’D2F3220 | CCATGGCATCTAGGTAAG | 3’D2R4093 | TGGCTGTTGGATTGAGAC | 874 | DENV2 |
| 18 | 5'D2F3096 | GCCAAAATCACACACCCTCT | 3'D2R4138 | GCCAGCTCCTTTTCTTGTTG | 1043 | DENV2 |
| 19 | 5'D2F3526 | TTGTTCCTGGAGGAAATGCT | 3'D2R4592 | CTGTAGGCTCCGTCTTCCAG | 1067 | DENV2 |
| 20 | 5’D2F3673 | GATGACATAGGTATGGGC | 3’D2R4389 | TATCGACATGCTACCATC | 717 | DENV2 |
| 21 | 5’D2F3953 | TGATATTACAAAATGCATGG | 3’D2R4853 | GTTTGGACGGCTCTTGGA | 901 | DENV2 |
| 22 | 5’D2F4408 | CAAACACTGACCATACTC | 3’D2R5275 | TGTGCTCAGCTCTGATGG | 868 | DENV2 |
| 23 | 5'D2F4488 | AGCATGGTACCTGTGGGAAG | 3'D2R5519 | ATGATTGGTGCATTGCTCTG | 1032 | DENV2 |
| 24 | 5’D2F4656 | ATTCCATACAATGTGGCA | 3’D2R5727 | CCAATCATTGGTTCTAGTCT | 1072 | DENV2 |
| 25 | 5'D2F4891 | GGTGCCGTATCTCTGGACTT | 3'D2R5995 | TGGCTTCTTTCCAGTGTGC | 1105 | DENV2 |
| 26 | 5’D2F5320 | ATGAGGCTGCTATCACCA | 3’D2R6023 | GGTGTGTTGATGTTATCT | 704 | DENV2 |
| 27 | 5'D2F5372 | ACGAAGCCCATTTCACAGAC | 3'D2R6454 | TGTCCAGTGCGTCTCTTGTC | 1083 | DENV2 |
| 28 | 5’D2F5618 | AAGCAGGAAATGATATAGCA | 3’D2R6374 | TTTCTTCC(A,G)GCTGCAAATTC | 757 | DENV2 |
| 29 | 5'D2F5831 | GTGAGGAGCGGGTGATTCT | 3'D2R6893 | GGTTGCTGGGTTGCAATACT | 1063 | DENV2 |
| 30 | 5’D2F6015 | CAATACACCAGAAGGAAT | 3’D2R6767 | TCTTGGGGTGTTCTCTGT | 753 | DENV2 |
| 31 | 5'D2F6264 | CTGGACAAAAGAAGGGGAAAG | 3'D2R7239 | CGCTGCTCTTTTCTGAGCTT | 976 | DENV2 |
| 32 | 5’D2F6487 | GGAAGGGCGTACAATCAT | 3’D2R7160 | AGAAGAGCTGCTGTGAGA | 674 | DENV2 |
| 33 | 5'D2F6665 | ACGCACAAATACAGCCACAC | 3'D2R7636 | TTTTTCCCAGTGCGTTCAGT | 972 | DENV2 |
| 34 | 5’D2F6895 | GAGAGCAACATCCTGGAC | 3’D2R7562 | GTGTTGGTTGTGTTCTTCAT | 668 | DENV2 |
| 35 | 5'D2F7028 | TAGCCAACCAAGCAACAGTG | 3'D2R8136 | TGAGGGCATATATGGGTTGAG | 1109 | DENV2 |
| 36 | 5’D2F7267 | GATGGAATAACAGTGATT | 3’D2R7823 | CCTCTGCCACAACCGAGG | 557 | DENV2 |
| 37 | 5’D2F7426 | ATCTCCACACTGTGGGAA | 3’D2R8237 | TTGGATACCCAGTACATC | 812 | DENV2 |
| 38 | 5'D2F7510 | TACTTGGCTGGAGCTGGACT | 3'D2R8666 | GGTTCTTGGGTTCTCGTGTC | 1157 | DENV2 |
| 39 | 5’D2F7830 | GTCATACTATTGTGGAGG | 3’D2R8615 | AATGGAGTCGTGTCTGTC | 786 | DENV2 |
| 40 | 5'D2F8025 | AAATGGAAGCACTGCAAAGG | 3'D2R8966 | CCGAATTCCCCTAGCTTCTT | 942 | DENV2 |
| 41 | 5’D2F8245 | GGGAACATAGTGTCATCA | 3’D2R9011 | GCTCCAAGCCACATGTACCA | 767 | DENV2 |
| 42 | 5'DF8545 | AGGCTGCTGACAAAACCTTG | 3'D2R9377 | TGCCCACTACCTCTTTGGTC | 833 | DENV2 |
| 43 | 5'D2F8792 | CCTTGGGAGCCATATTCACT | 3'D2R9773 | GGCTCTGCCAATCAGTTCAT | 982 | DENV2 |
| 44 | 5’D2F8888 | ATCTCCATCTTGAAGGAAAG | 3’D2R9665 | CCTCTTGAAGGTTCCCAT | 778 | DENV2 |
| 45 | 5'D2F9234 | CCACATGGAAGGAGAACACA | 3'D2R10167 | TGCTGCTTGGATGTTCTTTG | 934 | DENV2 |
| 46 | 5'D2F9543 | GGCCATCAGTGGAGATGATT | 3’D2R10723 | AGAACCTGTTGATTCAAC | 1181 | DENV2 |
| 47 | 5’D2F9697 | TCACACCATTTCCATGAG | 3’D2R10723 | AGAACCTGTTGATTCAAC | 1027 | DENV2 |
| 48 | 5'D2F10042 | GAAGACAAAACCCCAGTGGA | 3’D2R10723 | AGAACCTGTTGATTCAAC | 682 | DENV2 |
