# Supplementary material for: The origins of dengue and chikungunya viruses in Ecuador following increased migration from Venezuela and Colombia
Source: BMC Evol Biol. 2020 Feb 19;20:31. doi: 10.1186/s12862-020-1596-8 (PMC7031975; doi:10.1186/s12862-020-1596-8)
Supplement: Supplementary file 9 — Additional file 9. CHIKV Specific Primer Pairs Used on the Integrated Fluidic Circuits of the Access Array (Fluidigm) and Conventional PCR. [file 12862_2020_1596_MOESM9_ESM.docx]

Table S4: CHIKV Specific Primer Pairs Used on the Integrated Fluidic Circuits of the Access Array (Fluidigm) and Conventional PCR

| Primer Pairs | Forward  Primer | Sequences | Reverse  Primer | Sequences | Amplicon Size (bp) |
| --- | --- | --- | --- | --- | --- |
|  |  |  |  |  |  |
| 1 | 5’CHIKVF1 | ATGGCTGCKTGAGACACACGTA | 3'CHIKVR871 | ATGGAACACCGATGGTAGGTG | 871 |
| 2 | 5’CHIKVF1 | ATGGCTGCKTGAGACACACGTA | 3'CHIKVR1435 | CGGTACCACAAAGCTGTCAAAC | 1435 |
| 3 | 5'CHIKVF616 | AACCCCGTTCATGTACAATGC | 3’CHIKVR1435 | CGGTACCACAAAGCTGTCAAAC | 820 |
| 4 | 5'CHIKVF1317 | CACTGACCTGCTGCTGTCTATG | 3'CHIKVR2130 | AGTCCTGCAGCTTCTTCCTTC | 814 |
| 5 | 5’CHIKVF1469 | GGACTAGAATCAAGTGGTTGTTACGCAA | 3’CHIKVR2130 | AGTCCTGCAGCTTCTTCCTTC | 662 |
| 6 | 5'CHIKVF1908 | CAATCTCGCCTGAAGACTTCC | 3'CHIKVR2709 | TCCACTACAATCGGCTTGTTG | 802 |
| 7 | 5’CHIKVF2530 | GTGCGGCTTCTTCAATATGATG | 3’CHIKVR3343 | TCCAGGCCTATTATCCCAGTG | 814 |
| 8 | 5’CHIKVF2577 | AACATCTGCACCCAAGTGTACC | 3’CHIKVR3504 | GTCTCCTGTTGGCCGGTATAAT | 928 |
| 9 | 5'CHIKVF3332 | TAATAGGCCTGGAGGGAAGATG | 3'CHIKVR4134 | CTACGCACTCTTCATCGTTCTT | 803 |
| 10 | 5’CHIKVF3885 | GAACGAGTCATCTGCGTATTGG | 3’CHIKVR4725 | ATATCTCTGCCATATCCACTGC | 841 |
| 11 | 5'CHIKVF4450 | GAACCACCTCTTTACAGCCATGGA | 3'CHIKVR5266 | TACGGTGCTCATTACCCAGTCAGAC | 817 |
| 12 | 5’CHIKVF5065 | TGTACAGGAAGCGAGTACGACC | 3’CHIKVR5874 | TCTACTTTGCGCGACTGATACC | 810 |
| 13 | 5'CHIKVF5630 | ACGGACGACGAGTTACGACTAG | 3'CHIKVR6380 | CCCAGTATTCTTGGTTGCATG | 751 |
| 14 | 5’CHIKVF6241 | GAACACACTACAGAATGTACTGGCA | 3’CHIKVR6975 | AACAGAGTTAGGAACATACCGGATTTCATC | 735 |
| 15 | 5'CHIKVF6732 | TCATAGCCGCACACTTTAAGC | 3'CHIKVR7495 | AGGACCGCCGTACAAAGTTAC | 764 |
| 16 | 5’CHIKVF7200 | ATGATACTGTGACAGGAACAGCTTG | 3’CHIKVR8060 | GCGCATTCAAGGTCGTACTTAGAT | 861 |
| 17 | 5’CHIKVF7911 | CGAAGTCAAGCACGAAGGTAAGG | 3’CHIKVR8403 | GGAGCAGGGGAACGTGGTATTTG | 493 |
| 18 | 5'CHIKVF8329 | CCTGAGGGAGCCGAAGAGTGGA | 3'CHIKVR8945 | ACAGGAGGGTCGTGGTGAAATGG | 617 |
| 19 | 5'CHIKVF8819 | TTACTGGAACAATGGGACACTT | 3'CHIKVR9670 | TGAACGAGGCCACTGACACAACTA | 852 |
| 20 | 5’CHIKVF9550 | AAGTATTGGCCGCAGTTATCT | 3’CHIKVR10188 | TGTACCGCAGCATTTCACG | 639 |
| 21 | 5’CHIKVF9550 | AAGTATTGGCCGCAGTTATCT | 3’CHIKVR10627 | CTTTGCTCTCTCAGGCGTGCGACTTT | 1078 |
| 22 | 5’CHIKVF10445 | ACCATGCCGTCACAGTTA | 3’CHIKVR11296 | ACACGCATAGCACCACGATTAGAA | 852 |
| 23 | 5'CHIKVF11011 | CGGGAAGCTGAGATAGAAGTTGAA | 3'CHIKVR12036 | GAAATATTAAAAACAAAATAACATCTCCTACGTCCC | 1026 |
| 24 | 5’CHIKVF11278 | ATCGTGGTGCTATGCGTGTCGTTC | 3’CHIKVR12036 | GAAATATTAAAAACAAAATAACATCTCCTACGTCCC | 759 |
